# The Clinical Effectiveness of Web-Based Cognitive Behavioral Therapy With Face-to-Face Therapist Support for Depressed Primary Care Patients: Randomized Controlled Trial

**DOI:** 10.2196/jmir.2714

**Published:** 2013-08-05

**Authors:** Ragnhild Sørensen Høifødt, Kjersti R Lillevoll, Kathleen M Griffiths, Tom Wilsgaard, Martin Eisemann, Knut Waterloo, Nils Kolstrup

**Affiliations:** ^1^Department of PsychologyFaculty of Health SciencesUniversity of TromsøTromsøNorway; ^2^Centre for Mental Health ResearchThe Australian National UniversityCanberraAustralia; ^3^Department of Community MedicineFaculty of Health SciencesUniversity of TromsøTromsøNorway; ^4^Department of Community Medicine, General Practice Research UnitFaculty of Health SciencesUniversity of TromsøTromsøNorway

**Keywords:** cognitive therapy, therapy, computer-assisted, Internet, mental health, depression, randomized controlled trial, primary health care

## Abstract

**Background:**

Most patients with mild to moderate depression receive treatment in primary care, but despite guideline recommendations, structured psychological interventions are infrequently delivered. Research supports the effectiveness of Internet-based treatment for depression; however, few trials have studied the effect of the MoodGYM program plus therapist support. The use of such interventions could improve the delivery of treatment in primary care.

**Objective:**

To evaluate the effectiveness and acceptability of a guided Web-based intervention for mild to moderate depression, which could be suitable for implementation in general practice.

**Methods:**

Participants (N=106) aged between 18 and 65 years were recruited from primary care and randomly allocated to a treatment condition comprising 6 weeks of therapist-assisted Web-based cognitive behavioral therapy (CBT), or to a 6-week delayed treatment condition. The intervention included the Norwegian version of the MoodGYM program, brief face-to-face support from a psychologist, and reminder emails. The primary outcome measure, depression symptoms, was measured by the Beck Depression Inventory-II (BDI-II). Secondary outcome measures included the Beck Anxiety Inventory (BAI), the Hospital Anxiety and Depression Scale (HADS), the Satisfaction with Life Scale (SWLS), and the EuroQol Group 5-Dimension Self-Report Questionnaire (EQ-5D). All outcomes were based on self-report and were assessed at baseline, postintervention, and at 6-month follow-up.

**Results:**

Postintervention measures were completed by 37 (71%) and 47 (87%) of the 52 participants in the intervention and 54 participants in the delayed treatment group, respectively. Linear mixed-models analyses revealed a significant difference in time trends between the groups for the BDI-II, (*P*=.002), for HADS depression and anxiety subscales (*P*<.001 and *P*=.001, respectively), and for the SWLS (*P*<.001). No differential group effects were found for the BAI and the EQ-5D. In comparison to the control group, significantly more participants in the intervention group experienced recovery from depression as measured by the BDI-II. Of the 52 participants in the treatment program, 31 (60%) adhered to the program, and overall treatment satisfaction was high. The reduction of depression and anxiety symptoms was largely maintained at 6-month follow-up, and positive gains in life satisfaction were partly maintained.

**Conclusions:**

The intervention combining MoodGYM and brief therapist support can be an effective treatment of depression in a sample of primary care patients. The intervention alleviates depressive symptoms and has a significant positive effect on anxiety symptoms and satisfaction with life. Moderate rates of nonadherence and predominately positive evaluations of the treatment also indicate the acceptability of the intervention. The intervention could potentially be used in a stepped-care approach, but remains to be tested in regular primary health care.

**Trial Registration:**

Australian New Zealand Clinical Trials Registry: ACTRN12610000257066; http://apps.who.int/trialsearch/trial.aspx?trialid=ACTRN12610000257066 (Archived by WebCite at http://www.webcitation.org/6Ie3YhIZa).

## Introduction

### Overview

Depression is a highly prevalent disorder that often causes substantial functional impairment in daily life, reduction in quality of life, and increased medical service utilization [1-6]. There exist several effective psychological and pharmacological treatments for depression [7]. However, a large proportion of those suffering from this disorder receive inadequate treatment or no treatment at all [8,9]. Cognitive behavioral therapy (CBT) has proven to be as effective as pharmacotherapy in treating mild to moderate depression, with the benefit of reduced rates of relapse [10,11].

### Internet-Based Treatment of Depression

The principles and techniques of CBT have been extensively disseminated through self-help books and computer- or Internet-based programs. A substantive body of research shows that Internet-based CBT can be an efficacious treatment of depression (eg, [12-15]). Research also suggests that such interventions are cost-effective compared to face-to-face treatments because they result in symptom reduction and reduced burden of disease for patients and alleviate demands on clinician time and resources [16-18].

Self-help can be self-administered or guided by a therapist, although the active involvement of the therapist in guided self-help is less extensive than in conventional psychological therapy. Studies generally show small to moderate effects of self-administered, unguided CBT in the treatment of depression [19-23], although in some studies unguided interventions have yielded large treatment effects [24,25]. Still, an increasing amount of research has pointed to the importance of support in Internet-based interventions, with interventions offering some degree of support from a professional during treatment generally showing substantially larger treatment effects than interventions involving little or no professional support [26-28]. However, this conclusion is primarily based on meta-analytic results; the results from the few studies directly comparing guided and unguided interventions are mixed [14,24,29]. Overall, guided interventions show moderate to large treatment effects for depression, and the average effect sizes for guided self-help are comparable to the effects of time-limited face-to-face treatment (eg, [13-15,30]). This is further supported by a recent meta-analysis, which found no significant differences in effect between guided self-help and face-to-face therapy [31].

MoodGYM is a free Web-based CBT program developed to prevent and treat mild to moderate depression [32]. Studies have demonstrated the effectiveness of MoodGYM in reducing symptoms of depression and anxiety among public registrants, trial participants, callers to a national helpline service, users of the UK National Health Service portal, adolescent school-based populations, and in Australian and Norwegian student samples [20,24,25,29,33-35]. Positive effects have been shown to be sustained over 12 months [36]. However, few previous trials have investigated the effect of MoodGYM combined with therapist support. A study found that the conjunction of MoodGYM and face-to-face therapy was superior to both MoodGYM alone and for some outcome measures, to time-limited face-to-face therapy alone [29]. Also the results of a cluster-randomized trial suggested positive effects of the combination of MoodGYM and support from general practitioners (GPs) compared to GP care alone [37].

### Depression Treatment in Primary Health Care

Most patients with psychological problems will receive most or all of their mental health care in primary care, and findings suggest that many patients prefer to consult their GP for treatment of depression [2,38-40]. Clinical practice guidelines primarily recommend treating mild to moderate depression using psychosocial interventions [41,42], and this is also in accordance with reported patient preferences [43-45]. Nevertheless, structured psychological interventions are infrequently delivered in general practice [46-48] because of time constraints [49-51] and a lack of knowledge and competence among GPs in the delivery of evidence-based psychological interventions [51,52]. The use of CBT-based self-help resources could be a way to improve the delivery of psychological interventions in general practice. This would allow for short consultations and for the clinician to be a facilitator rather than a cognitive therapist. These features could improve feasibility in general practice, where the volume of patients is high and it is essential that interventions are brief and practical.

### Aim of the Study

The current study was designed to trial a procedure for depression treatment that could be suitable for implementation in general practice. The project was planned as the first phase of research for this treatment, with the second phase focusing on further evaluation carried out in everyday general practice. The aim was to evaluate the effectiveness and acceptability of a guided self-help intervention combining the MoodGYM program with brief face-to-face therapist support in a sample of primary care patients with mild to moderate symptoms of depression. This was investigated in a randomized controlled trial comparing the guided self-help intervention to a delayed-treatment control condition. The primary hypothesis was that therapist-supported Web-based CBT would lead to a larger reduction in depressive symptoms than the control condition. To determine if the intervention was acceptable to patients, satisfaction with treatment, adherence, and reasons for dropout were investigated.

## Methods

### Study Design

The study was a randomized controlled trial with balanced randomization (1:1). Participants were randomly allocated to a treatment condition comprising 6 weeks of Web-based CBT with therapist support, or to a 6-week waitlist for the same treatment during which time they could also access treatment as usual. The study was conducted at the Department of Psychology at the University of Tromsø where a small self-help outpatient clinic was established. The research protocol was approved by the Regional Committee for Research Ethics in Northern Norway (2011/2163) and the Human Ethics Committee of the Australian National University (ANU). The trial was registered in the Australian New Zealand Clinical Trials Registry (ACTRN12610000257066). The trial is reported in accordance with the CONSORT-EHEALTH [53] (see Multimedia Appendices 1-3).

### Participants

Participants (N=106) were recruited between October 2010 and October 2012 from GPs, primary care nurses, and from waitlists of primary care referrals at 2 psychiatric outpatient clinics. Calculations of required sample size were based on a power of .80, significance level of .05 (2-sided), and an expected effect size of 0.6 on depressive symptoms at posttest. The estimations necessitated a sample size of 45 participants per group. A median dropout rate between 17% and 19% has been reported for computerized or Web-based treatment programs [54,55]. With a 20% expected dropout, a total sample size of 108 was required to gain sufficient power, yielding group sizes of 54 participants.

Local GPs and primary care nurses were informed about the study both verbally at practice meetings and through written information. They provided patients who they considered as mildly to moderately depressed based on clinical appraisal and/or screening instruments with written information about the project. Potentially eligible patients on waitlists for psychiatric outpatient treatment were identified by clinic staff and subsequently received information by postal mail from the research group. When informing a patient of the project, all recruiters were asked to send a notification to the research group by using a prepaid envelope. The notification simply notified the researchers that a patient had been informed of the project and did not reveal any information about the patient. Patients were provided with general information about the treatment and the aim of the project and detailed information about the methods for handling issues of privacy and anonymity. They were informed that they could expect to commence treatment within 6 weeks of the initial contact. To participate, patients sent in a signed informed consent form providing contact details. Study inclusion criteria were: (1) age 18-65 years, (2) access to the Internet, and (3) a score between 14 and 29 on the Beck Depression Inventory-II (BDI-II), indicating mild to moderate symptoms of depression. During the first months of the study, the protocol was changed by extending the inclusion criterion on the BDI-II to include participants with scores between 10 and 40. This change was because of insufficient recruitment and the clinical appraisal that patients with scores above 30 could possibly benefit from the treatment based on their daily functioning and motivation. In addition, their depression was too mild to assure them other public treatment options. Furthermore, several patients with a BDI-II score below 14 reported a need for treatment. Based on this revised criteria, 7 eligible patients were falsely excluded in the initial phase of the trial. Individuals currently undergoing CBT were excluded, whereas individuals who used antidepressant medication were stabilized for 1 month prior to evaluation of diagnostic eligibility. To maximize the external validity of the trial, a heterogeneous group of patients with depressive symptoms was included, independent of a particular diagnosis. Therefore, medical or psychiatric comorbidities only restricted inclusion when there was a need for immediate treatment of these comorbid conditions (suicidal ideation, current psychosis) or if the conditions were expected to markedly interfere with treatment of the depressive condition (alcohol or drug use disorders).

### Measures

All outcome measures were based on self-report. Assessments of symptoms of depression, anxiety, and quality of life using paper-and-pencil questionnaires were completed by all participants at baseline, posttreatment, and at 6 months posttreatment (online questionnaires). The control group also completed these inventories before entering online treatment (postwaiting). BDI-II was administered before every consultation during the intervention phase.

The primary outcome measure was the BDI-II, a 21-item measure of severity of depressive symptoms during the past 2 weeks [56]. Each item is rated on a 4-point scale ranging from 0 to 3. Studies consistently support the BDI-II as a reliable, internally consistent, and valid scale for assessing depression both in psychiatric outpatients, the general population, and in primary care settings [56-58]. Several studies have found high correlations between the paper-and-pencil and the computerized/online versions of the BDI-II [59-62]. In the present study, internal consistency (Cronbach alpha) was .78 pretreatment, .93 posttreatment, and .94 at 6-month follow-up, respectively.

The secondary outcome measures consisted of the Beck Anxiety Inventory (BAI), the Hospital Anxiety and Depression Scale (HADS), and 2 measures of quality of life—Satisfaction With Life Scale (SWLS) and the EuroQol Group 5-Dimension Self-Report Questionnaire (EQ-5D)—as well as a measure of treatment satisfaction. The quality of life measures were included during the initial phase of the trial considering that the extension of outcomes beyond symptom measures would strengthen the study.

The BAI is a 21-item measure of anxiety symptom severity [63]. Each item is rated from 0 to 3 depending on symptom severity during the past week. The inventory possesses high internal consistency and reliability, as well as robust convergent and discriminant validity [64-66]. Equivalent psychometric properties have been shown across paper-and-pencil and online formats of the questionnaire, and the 2 formats are highly correlated [59,67]. Cronbach alphas in the present study were .93 at pretreatment, .88 at posttreatment, and .92 at 6-month follow-up, respectively.

The HADS is a 14-item inventory with 2 subscales of 7 items each, measuring depression and anxiety, respectively [68]. Each item is rated on a 0 to 3 scale. The inventory has good to very good construct validity and internal consistency [68-70]. Most factor analyses confirm a 2-factor solution comprising a depression and an anxiety subscale [69]. Paper-and-pencil and Internet administration of the measure yields comparable psychometric properties, but Internet administration may overestimate scores [70,71]. In the present study, Cronbach alpha was .68 and .82 at pretreatment, .82 and .84 at posttreatment, and .87 and .86 at 6-month follow-up for the depression and anxiety subscales, respectively.

The EQ-5D is a generic questionnaire evaluating health-related quality of life [72]. The respondent marks his/her level of functioning (no problems, some problems, extreme problems) for each of 5 dimensions (mobility, self-care, usual activities, pain/discomfort, and anxiety/depression) and rates his/her health state on a visual analog scale (EQ VAS) with the endpoints labeled best imaginable health state and worst imaginable health state, respectively. For the 5 dimensions, a scoring algorithm (the MVH_A1 tariff) based on preference weights was used to aggregate an index score (EQ Index) [73]. The health states “perfect health” (no problems on any dimension) and “dead” are assigned the values of 1 and 0, respectively. The instrument has been demonstrated to discriminate between subgroups of patients with differing severities of mental illness and to capture changes in quality of life associated with improved mental health over time [74]. No significant differences have been found between scores obtained using paper and computerized modes of administration [75,76].

The SWLS measures global life satisfaction as a cognitive-judgemental process, in which individuals assess their quality of life according to their own criteria [77]. The respondent rates on a 7-point Likert scale the degree to which he/she agrees with 5 statements. Several studies confirm the scale’s unidimensional structure and support its sound psychometric properties, including internal consistency, test-retest reliability, as well as convergent and discriminant construct validity [78-80]. Research indicates that Internet data on this measure is as reliable and valid as paper-and-pencil data [81]. Cronbach alpha in this study was .79 at pretreatment, .87 at posttreatment, and .93 at 6-month follow-up.

The Mini-International Neuropsychiatric Interview (MINI) is a short, structured diagnostic interview for identifying the diagnoses of the *Diagnostic and Statistical Manual of Mental Disorders* (Fourth Edition; *DSM-IV*) and the *International Classification of Diseases, Tenth Revision* (*ICD-10*). It consists of 17 modules. A comparison of MINI with other structured clinical interviews shows sensitivities and specificities above 0.70 for most diagnoses [82]. Excellent interrater reliability has been reported. The MINI Interview was used to determine psychiatric comorbidity and for excluding participants with current psychosis or suicidal ideation, as indicated by 17 points or more on the suicidal ideation module.

The Alcohol Use Disorders Identification Test (AUDIT) is a screening instrument consisting of 10 questions about alcohol use in the past 12 months, alcohol dependence symptoms, and alcohol-related problems [83]. Eight items are rated on a 5-point scale (0-4) and 2 items are rated on a 3-point scale (0, 2, 4). A large body of research confirms the favorable internal consistency, reliability, and criterion validity [83-85]. In this study, the scale was used to screen for alcohol use problems. A cutoff score of 20 was chosen to exclude patients in need of further diagnostic evaluation for alcohol dependence [85]. For participants scoring above16, alcohol use was monitored during treatment.

The Drug Use Disorders Identification Test (DUDIT) is an 11-item screening instrument measuring patterns of substance use during the past 12 months, as well as various drug-related problems [86]. Nine items are rated on a 5-point scale (0-4) and 2 items are rated on a 3-point scale (0, 2, 4). In a sample of drug users, the scale has shown good reliability, and it predicts drug dependence with a sensitivity of 0.90 and average specificity of approximately 0.80 [86]. In the present study, the DUDIT was used to screen for drug use disorder. A cutoff score of 25 was used to exclude patients with a high probability of drug dependency.

Satisfaction with treatment was measured by 9 questions that respondents rated on a 5-point scale (1-5, very negative to very positive). The questions concerned their satisfaction with the intervention as a whole and various aspects of the self-help program and follow-up sessions. The general content of the questions was influenced by patient satisfaction questionnaires used in other studies [87-89]. However, the exact content was tailored to tap into aspects of treatment considered important for the purpose of the present investigation. The questions are described in detail in the Results section.

Treatment variables included module completion, number of sessions, treatment duration, session duration (in minutes, not including screening), and total time spent by therapists (time spent outside the consultations was not registered). User data on module completion was registered online and was denoted by a number between 0 and 4, with 0 indicating no use and 4 indicating completion of the module. For the variable time spent by therapists, the amount of missing data was considerable (51.9% for time spent on screening, 14.6% for total time); thus, these data can only be considered estimates. Total time was estimated by imputing mean screening time for missing data concerning screening duration and each individual’s mean session duration for missing data from treatment sessions.

### Procedure

After informed consent was obtained, participants were screened for inclusion through a face-to-face session. A computerized random number generator randomized identification (ID) numbers to the 2 groups (generated by KL). Eligible participants were given ID numbers following a chronological sequence. To ensure equal group sizes, blocked randomization with variable block sizes was used. Patients could not be blinded to group assignment, but were blinded to the status of the waitlist as a control condition.

Screening, enrollment, and treatment were carried out by 2 licensed clinical psychologists (RSH and KL) with basic CBT skills and good knowledge of the MoodGYM program. Both had less than 2 years of experience in clinical practice and no prior experience with Internet-based treatment. The therapists were not blind to the participants’ group allocation. However, steps were taken to blind the evaluation of outcomes by ensuring that posttests were performed by a research assistant unaware of the participants’ allocation assignment.

### Intervention

Participants in both groups were free to access usual primary care treatment, which could include antidepressant medication, informal supportive therapy, or referral to specialist mental health services.

**Figure 1:**
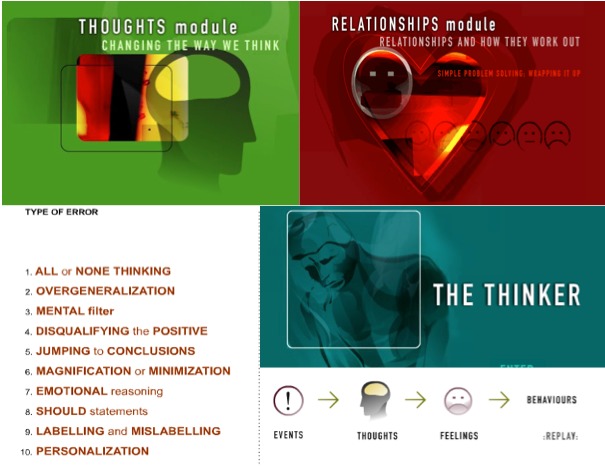
Screenshots from MoodGYM.

The guided self-help intervention involved 3 components: (1) The Norwegian version of the Web-based CBT program MoodGYM version 3 [90], (2) brief face-to-face therapist support, and (3) tailored emails between sessions. MoodGYM was originally developed at the Australian National University to prevent depression in young people aged between 15 and 25 years. However, data from individuals who used the English version of the program has shown that most users were aged 25 to 44 years, and that the users’ average depression and anxiety scores were elevated compared to the general population [91]. Therefore, the program appears useful for older age groups than originally targeted, and for individuals with elevated levels of depressive and anxious symptomatology. The program consists of 5 modules and a personal workbook containing exercises and assessments. Module 1 through 3 focus on the cognitive model, typical patterns of dysfunctional thinking, and exercises to identify and restructure dysfunctional thinking, as well as behavioral strategies to increase engagement in positive activities. Module 4 focuses on stress and stress reduction and introduces relaxation techniques. Module 5 covers simple problem solving and typical responses to broken relationships. Each module takes approximately 45 to 60 minutes to work through. See Figure 1 for screenshots from the program.

In the first session after screening, participants were introduced to the program, received their trial username and password, and were instructed to work at home with 1 module each week. After each module, participants received face-to-face support (15-30 minutes). The therapists followed a guideline script with 3 compulsory topics for every consultation: (1) monitoring of depression symptoms and discussion of changes, (2) a focus on the important topics and exercises covered by each module and the participants’ experiences of working with it, and (3) introduction of the next module and motivate participants to adhere to the treatment plan. The main focus of the therapist was on reinforcing the efforts made by participants and helping them to relate to the material and to incorporate the use of techniques from the program into their everyday living. If time permitted, participants could also bring up other topics they considered important in relation to their depression. In the concluding session, the experiences and outcomes of treatment were discussed. Therapists aimed to meet participants weekly and to complete the intervention over 7 weeks. However, the interval between sessions and the number of sessions were allowed to vary somewhat to provide flexibility in meeting individual needs. Between sessions, participants received tailored emails aiming to motivate them to work with the self-help program. The emails introduced the next module, and some contained brief advice on how to overcome depressive symptoms (eg, the importance of behavioral activation). Participants did not get a mental health record at the clinic, but a short case summary was sent to their GP (with consent from the participants). Participants considered in need of more extensive treatment throughout or after completing the trial were assisted in the process of referral to specialized mental health services. The Web-based program did not store any personally identifying information about users.

### Statistical Analyses

All analyses were carried out using IBM SPSS Statistics version 19 for Windows (IBM Corp, Armonk, NY, USA), except for the power calculation which was performed using G*Power (Heinrich-Heine-Universität, Institut für Experimentelle Psychologie, Düsseldorf, Germany).

Differences between the groups on baseline characteristics were examined by performing chi-square tests for categorical variables and 1-way analysis of variance (ANOVA) for continuous variables. A logistic regression analysis with backward stepwise method was used to explore whether missing data at postintervention (not completing postintervention measures) could be significantly predicted by participant characteristics.

Results on the BDI-II and BAI were analyzed using intention-to-treat (ITT) analyses in which participants are analyzed in the group they were randomized to, irrespective of treatment adherence. Because of missing data at pretest for the remaining secondary measures (3% missing on the HADS, and 17% and 19% missing on SWLS and EQ-5D, respectively), modified ITT analysis was performed including all participants completing the measures at least once. Effects were tested by performing linear mixed-models analysis using the restricted maximum likelihood (REML) estimation procedure and an unstructured covariance matrix. Since linear mixed-models analysis can handle incomplete data, no procedure for imputation of missing data was utilized in the analysis [92]. For the analysis of BDI-II during the treatment phase, random intercepts across participants were estimated, and BDI-IIs from every treatment session were included for the intervention group. Time was coded as 0 for baseline and as number of weeks from baseline for all subsequent measures. To control for differences in treatment duration, the time frame was made comparable between groups by including only measures up to 7 weeks after baseline (the planned time frame for completing the intervention) for the intervention group in the main analysis. For the secondary measures and for the analysis including the 6-month follow-up data on the BDI-II, repeated measures linear mixed-models analysis was performed with occasion (baseline, posttest, 6-month follow-up) as the repeated factor. This procedure was deemed acceptable because linear regression analyses did not find treatment duration to be a significant predictor of symptom change during the treatment phase of the intervention group for any of the secondary measures (beta=-.16 to .28, *t*_*29-35*_=–0.91 to 1.58, *P*=.12-.92). Scores on the last BDI-II from participants completing 5 or more weeks of treatment but missing formal posttest data (n=8), were included in the analysis, because this was considered to give a more accurate estimate of change over time.

For completers, analyses of covariance (ANCOVA) were performed with postsymptom scores as the dependent variable and preintervention symptom scores and treatment duration as covariates. Effect sizes (Cohen’s *d*) were calculated for within- and between-group changes based on estimated means or beta coefficients [93]. For the ITT analyses, calculations were based on pooled standard deviations calculated from the square root of each group’s variance parameters from the mixed models analysis: the single variance estimate of the procedure with a random intercept, and the sum of the variance estimates at each time point of interest minus 2 times the covariance estimate between these time points for the repeated procedure [94,95]. For the completer analyses, the square root of the mean square error, equivalent to the pooled standard deviation, was used. A Cohen’s *d* of 0.2 was considered a small effect, 0.5 a medium effect, and 0.8 or more a large effect [96].

Clinically significant changes on the BDI-II were assessed using the criteria for reliable change and cutoff points developed for the BDI by Seggar et al [97], based on the definition by Jacobson and Truax [98]. Recovery was defined as the combination of reliable improvement (a change of more than 8.5 points on the BDI-II) and endpoint symptom level below the clinical cutoff of 14.3. For those with subclinical symptoms at baseline, reliable improvement required a change of more than 4.6 points and recovery required reliable improvement plus an endpoint less than 4.1 [97]. Change scores between baseline and 7 weeks of treatment were used for the intervention group.

## Results

### Sample Characteristics

**Figure 2:**
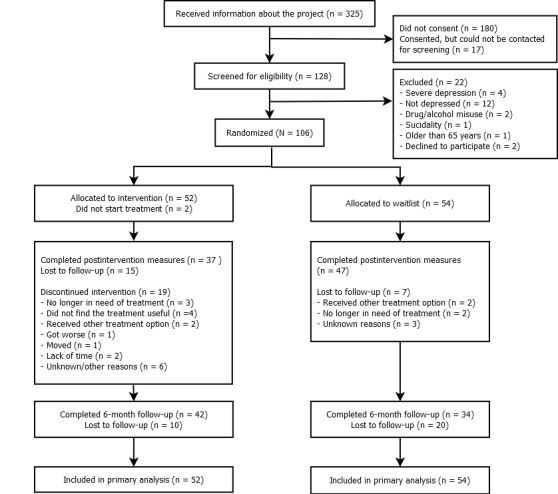
Flow of participants through the trial.

Figure 2 shows the flow of participants through the trial. Of the 128 individuals screened for participation, 106 (83%) were found eligible. Most participants, including 49 of 52 (94%) in the intervention group and 48 of 54 (89%) in the delayed-treatment group, were recruited from GPs. The remaining participants were recruited from waitlists at 2 outpatient clinics (n=3), from primary care nurses (n=4), and from a low-threshold clinic for youth (n=2). Postintervention measures were completed by 71% (37/52) and 87% (47/54) of the participants in the intervention and the delayed-treatment group, respectively. The 6-month follow-up assessment was completed by 42 participants (81%) in the intervention group and by 34 participants (63%) in the control group.

Group and educational level emerged as significant predictors of dropout at postintervention. The odds of dropping out before the posttest was significantly higher for participants in the intervention group relative to participants in the delayed-treatment group (OR 3.03, 95% CI 1.08-8.47, *P*=.04), and significantly lower for participants with higher education relative to participants with a lower educational level (OR 0.36, 95% CI 0.13-0.99, *P*=.048). No other demographic or clinical variables predicted dropout at postintervention.

Table 1 shows the descriptive characteristics of the sample. For most demographic and clinical variables the 2 groups did not differ significantly at baseline (*P*=.10-.90). However, the groups differed significantly with regard to age (*P*=.045), with the intervention group being slightly older than the control group. The groups also differed on the variable comorbid anxiety (*P*=.03), with the number of participants with an anxiety disorder being significantly higher in the control group compared to the intervention group. Baseline scores on all symptom and outcome measures were comparable between the groups (*P*=.13-.87).

Further exploration of the 2 groups with regard to anxiety level as measured with BAI shows that although a higher proportion of the control group had symptoms corresponding to moderate or severe anxiety compared to the intervention group, the distribution across the categories minimal, mild, moderate, and severe anxiety did not differ significantly (*P*=.29).

**Table 1:** Participant characteristics at baseline.

Participant characteristics		Intervention (n=52)	Waitlist (n=54)	Total (N=106)
**Gender, n (%)**				
	Male	15 (28.8)	14 (25.9)	29 (27.4)
	Female	37 (71.2)	40 (74.1)	77 (72.6)
**Age (years)**				
	Mean (SD)	38.3 (12.2)	33.9 (9.9)	36.1 (11.3)^a^
	Range	19 - 63	18 - 58	18 - 63
**Marital status, n (%)**				
	Married/living together	28 (53.8)	31 (57.4)	59 (55.7)
	Separate living	2 (3.8)	4 (7.4)	6 (5.7)
	Divorced	3 (5.8)	5 (9.3)	8 (7.5)
	Single	19 (36.5)	14 (25.9)	33 (31.1)
**Highest educational level,** ^b^ **n (%)**				
	Compulsory school (9 or 10 years)	6 (11.5)	1 (1.9)	7 (6.6)
	High school	16 (30.8)	26 (48.1)	42 (39.6)
	University, 3 years	17 (32.7)	14 (25.9)	31 (29.2)
	University, ≥5 years	13 (25.0)	12 (22.2)	25 (23.6)
**Employment, n (%)**				
	Employed (full- or part-time)	37 (71.2)	37 (68.5)	74 (69.8)
	Student	5 (9.6)	6 (11.1)	11 (10.4)
	Long term sick	7 (13.5)	3 (5.6)	10 (9.4)
	Homemaker	1 (1.9)	5 (9.3)	6 (5.7)
	Unemployed	2 (3.8)	3 (5.6)	5 (4.7)
	Sick leave (employed sample)	18 (48.6)	21 (56.8)	39 (52.7)^c^
**Present treatment,** ^d^ **n (%)**				
	Medication	13 (25.0)	7 (13.0)	20 (18.9)
	Other treatment	7 (13.5)	4 (7.4)	11 (10.4)
	None	36 (69.2)	44 (81.5)	80 (75.5)
**Treatment history,** ^e^ **n (%)**				
	Earlier	32 (61.5)	30 (55.6)	62 (58.5)
	None	18 (34.6)	23 (42.6)	41 (38.7)
Depression current,^f^n (%)		26 (50.0)	25 (46.3)	51 (48.1)
**Number of major depressive episodes,** ^g^ **n (%)**				
	0	5 (9.6)	6 (11.1)	11 (10.4)
	1	16 (30.8)	18 (33.3)	34 (32.1)
	2 - 4	14 (26.9)	15 (27.8)	29 (27.4)
	≥5	14 (26.9)	11 (20.4)	25 (23.6)
**Comorbidity,** ^h^ **n (%)**				
	Anxiety	12 (23.1)	23 (42.6)	35 (33.0)^a^
	Other axis-I disorder	1 (1.9)	4 (7.4 %)	5 (4.7)
**Symptom measures,** ^i^ **mean (SD)**				
	AUDIT	4.8 (3.8)	5.4 (4.3)	5.1 (4.0)
	DUDIT	0.4 (2.2)	0.7 (2.5)	0.6 (2.3)
**Internet Use,** ^j^ **n (%)**				
	Daily	50 (96.2)	42 (77.8)	92 (86.8)
	Weekly	0 (0.0)	3 (5.6)	3 (2.8)

^a^ *P*<.05.

^b^0.9% missing.

^c^36.8% of the total sample.

^d^Medication is antidepressants; other treatment is psychological therapy other than CBT.

^e^2.8% missing.

^f^Major depressive episode fulfilling *DSM-IV* criteria.

^g^6.6% missing.

^h^Anxiety includes panic disorder, agoraphobia, social phobia, and generalized anxiety; other axis-1 disorders include bipolar disorder, obsessive-compulsive disorder, bulimia, and posttraumatic stress disorder.

^i^AUDIT: Alcohol Use Disorders Identification Test, 0.9% missing; DUDIT: Drug Use Disorders Identification Test, 0.9% missing.

^j^10.4% missing.

### Attrition and Adherence

Of the 52 participants in the intervention group, 31 (60%) adhered to the treatment program in that they completed MoodGYM and attended at least 7 sessions. Total nonadherence was 40% (21/52). Reasons for nonadherence are summarized in Figure 2. Overall, the sample starting treatment (n=50) completed a mean of 3.8 (SD 1.7) of the 5 modules, attended a mean of 7.2 (SD 2.3) sessions, with average session duration of 27.7 (SD 6.2) minutes. The average number of weeks in treatment was 11.3 (SD 7.2). Total time spent by therapists ranged from 70 to 506 minutes (mean 242.1, SD 96.6). A previous study found that versions of the MoodGYM program encompassing Module 2 (extended CBT) were associated with greater improvements than versions excluding this module [91]. This suggests that Module 2 may be a particularly important treatment component. Of the 50 participants starting treatment, 86% (n=43) completed 2 or more modules, indicating that they may have completed enough of the treatment program to generate beneficial outcomes.

### Depression and Anxiety Symptoms

Table 2 depicts the preintervention, postintervention, and 6-month follow-up means and standard deviations for each group, as well as within-group and between-group effect sizes. The ITT analysis for the primary outcome measure, BDI-II, revealed a significant time by treatment group interaction (*F*_1,244.83_=9.55, *P*=.002, *d*=0.65). There was also a significant effect of time (*F*_1,245.37_=11.87, *P*=.001). Both groups experienced significant improvements of depressive symptoms during the intervention phase, but this improvement was significantly larger in the intervention group compared to the delayed-treatment group. Because most of the intervention group had not yet completed treatment at 7 weeks, the analysis was repeated using scores from the intervention group up to 8, 10, 12, and 14 weeks. The interaction term remained significant (*F*_1,268.81-333.62_=6.34-10.88, *P*=.01-.001, *d*=0.54-0.67). Repeated linear mixed-models analysis also found significant different time trends for the groups between posttest and 6-month follow-up (*t*_81.17_=2.88, *P*=.005). During this time, the delayed-treatment group had received treatment, and pairwise comparisons indicated a significant decrease in symptoms in this group (*P*=.001), whereas level of depressive symptoms in the intervention group remained stable (*P*=.56).

The ITT analysis for the BAI revealed no significant interaction between treatment group and occasion (*F*_1, 84.31_=0.37, *P*=.69). Pairwise comparisons showed that both groups improved significantly between baseline and posttest (*P*=.007 and *P*=.02 for the intervention and control group, respectively; see Table 2). Between posttest and 6-month follow-up, the control group further improved (*P*=.04), whereas the intervention group remained unchanged (*P*=.36). Analyses for the HADS subscales yielded significant group by occasion interactions for both the depression subscale (*F*_1,78.05_=14.68, *P*<.001) and the anxiety subscale (*F*_1,78.07_=8.10, *P*=.001). Pairwise comparisons for both subscales found that the intervention group, but not the control group, reduced their scores significantly between pretest and posttest (*P*<.001 and *P*=.46, respectively, for the depression subscale and *P*<.001 and *P*=.44, respectively, for the anxiety subscale; Table 2). Between posttest and 6-month follow-up, the control group experienced a significant reduction in both depressive and anxiety symptoms (*P*=.001 and *P*<.001, respectively), whereas scores did not change significantly for the intervention group for the depressive subscale (*P*=.07) or for the anxiety subscale (*P*=.80).

For all reported ITT and modified ITT analyses, baseline anxiety symptoms on the BAI and age were included as covariates, with the exception that baseline BAI scores were not included as a covariate in the analysis of BAI. The effect of BAI scores was consistently significant (*P*=.02 to *P*<.001). The effect of age was significant in 2 of 8 models (*P*=.008-.03).

#### Completer Analyses

The results of the ANCOVA revealed the same pattern of results. There was a significant effect of group on posttest level of depressive symptoms measured with BDI-II (n=84; *P*<.001, *d*=1.09) and with HADS (n=76; *P*=.002, *d*=0.95), and level of anxious worry as measured with HADS (n=76; *P*=.002, *d*=0.97) after controlling for the effect of baseline symptoms and treatment duration. The results were not significant for anxiety as measured with BAI (n=83; *P*=.47, *d*=0.20). Thus, for depression and anxiety measured with HADS, the groups differed significantly in posttest scores after controlling for differences in preintervention scores and treatment duration. Baseline symptoms were significantly related to posttest symptoms for all measures (*P*<.001), whereas treatment duration did not significantly affect outcome (*P*=.19-.92).

### Quality of Life

For the SWLS, the modified ITT analysis showed a significant interaction between treatment group and occasion (*F*_1,75.75_=8.49, *P*<.001). Pairwise comparisons found a significant increase in satisfaction for the intervention group from pretest to posttest (*P*<.001), but no such change for the delayed-treatment group (*P*=.52; see Table 2). Between posttest and 6-month follow-up, there was a significant increase in life satisfaction in the control group (*P*=.01), whereas the intervention group did not experience significant changes (*P*=.08). The analyses for EQ Index yielded an overall significant difference in time trends between the groups (*F*_1,67.42_=3.55, *P*=.03). Between pretest and posttest, there was no significant interaction between treatment group and occasion (*t*_68.82_=–1.00, *P*=.32), with both groups improving at comparable rates over time. However, there was a significant group by occasion interaction between posttest and 6-month follow-up (*t*_63.96_=–2.66, *P*=.01, with the control group showing significant improvement (*P*=.02), whereas there were no significant changes in the intervention group (*P*=.18). For the EQ VAS, the overall interaction between group and occasion was not significant (*F*_1,71.32_=2.25, *P*=.11). Between pretest and posttest both the intervention group (*P*<.001) and the control group (*P*=.03) experienced significantly improved self-reported health state (Table 2), but the interaction between group and occasion was not significant (*t*_66.07_=–1.94, *P*=.06). Between posttest and 6-month follow-up, pairwise comparisons showed a slight, but nonsignificant, improvement in the control group (*P*=.06), whereas scores in the intervention group remained stable (*P*=.74).

#### Completer Analyses

Similar results were found for the completer analyses, in which ANCOVA showed a significant effect of group for the SWLS (n=66) after controlling for baseline levels of life satisfaction and treatment duration (*P*=.006, *d*=0.86). There was no significant effect of group on health-related quality of life at posttest, EQ Index (n=63; *P*=.56, *d*=0.18), or health state at posttest, EQ VAS (n=61; *P*=.11, *d*=0.52), despite a moderate effect size for the latter. The effect of baseline scores was significant for all measures (*P*≤.002). There were no significant effects of treatment duration (*P*=.14-.99).

### Clinical Significance of Changes in Depressive Symptoms

Table 3 presents data on clinically significant change on the BDI-II, based on scores at 7 weeks for the intervention group. The ITT procedure was used (classifying all who did not start treatment or did not complete their waitlist period as nonresponders). The results of the chi-square tests for the full sample and the sample with BDI scores above clinical cutoff show that significantly more participants recovered in the intervention group compared to the delayed-treatment control group. Conversely, a significantly smaller proportion of the intervention group experienced no change within the intervention period. For the sample fulfilling the criteria for a major depressive episode at baseline, the same trend was evident, but the difference in rate of recovery did not reach significance. The rates of improvement and deterioration were similar in the 2 groups for all analyses. The same analysis carried out after excluding participants (n=8) who during the waitlist or intervention period started or increased their dosage of antidepressant medication, or commenced other psychological treatment, produced similar patterns of results. At 7 weeks of treatment 37 of 52 participants (71%) in the intervention group had completed 2 or more modules, whereas 15% (n=8) had completed treatment.

**Table 2:** Estimated means (EM),^a^ observed means (OM), observed standard deviations (SD), standard deviations based on linear mixed-models variance estimates (SDm), and effect sizes from pretreatment (pre) to posttreatment (post) and pretreatment to 6-month follow-up (6 m) for the intervention and the delayed-treatment control group.

Measures		Intervention (n=52)				Delayed treatment (n=54)				Effect size (Cohen’s *d*)^b^					
		EM	OM	SD	SDm	EM	OM	SD	SDm	Pre-post			Pre-6 m		
										B	Wi	Wdt	B	Wi	Wdt
**BDI-II**										0.65	–0.98	–0.65	–0.12	–0.81	–1.02
	Pretreatment	21.37	21.13	6.85		21.85	22.27	6.74							
	7 weeks	15.15^c^	14.20^c^	8.15	5.86	19.07	18.63	8.64	4.63						
	6 months	13.39	12.45	9.32	10.35	11.86	12.82	10.98	9.56						
**BAI**										0.08	–0.41	–0.35	–0.12	–0.48	–0.65
	Pretreatment	12.23	12.05	11.10		15.10	15.33	10.90							
	Posttreatment	8.80	8.36	9.26	8.21	12.30	12.83	8.10	8.03						
	6 months	7.46	7.07	6.61	10.37	9.19	9.41	9.79	9.05						
**HADS Depression**										1.10	–1.17	–0.11	–0.10	–0.53	–0.65
	Pretreatment	8.21	8.08	2.92		7.40	7.61	3.13							
	Posttreatment	4.67	4.24	2.61	3.13	7.07	7.19	3.63	2.70						
	6 months	5.91	5.76	4.05	4.29	4.66	4.85	3.77	4.16						
**HADS Anxiety**										0.74	–0.60	0.13	–0.13	–0.52	–0.55
	Pretreatment	9.14	8.81	3.95		9.15	9.59	4.59							
	Posttreatment	7.15	6.74	3.69	3.29	9.52	10.07	4.18	3.04						
	6 months	6.99	6.57	4.16	3.99	6.40	6.94	4.79	4.88						
**SWLS**										0.85	0.85	0.12	–0.12	0.35	0.52
	Pretreatment	16.41	16.54	5.25		16.83	16.36	5.75							
	Posttreatment	20.38	21.46	6.04	4.60	17.28	17.21	5.02	3.69						
	6 months	18.66	19.00	6.63	6.42	19.79	20.00	6.91	5.52						
**EQ-5D VAS**										0.47	0.79	0.42	–0.01	0.71	0.55
	Pretreatment	58.59	59.13	18.68		56.01	54.93	17.87							
	Posttreatment	71.13	73.88	15.30	16.35	61.67	60.77	17.09	13.10						
	6 months	70.12	71.79	14.65	16.04	67.78	66.41	20.86	21.56						
**EQ-5D Index**										0.22	0.59	0.26	–0.33	0.26	0.74
	Pretreatment	0.63	0.63	0.23		0.61	0.60	0.25							
	Posttreatment	0.75	0.80	0.17	0.24	0.68	0.67	0.24	0.27						
	6 months	0.70	0.72	0.26	0.30	0.78	0.77	0.20	0.21						

^a^Estimated means (except for BAI) are adjusted for the covariates baseline BAI score and age.

^b^B: between-group effect size; Wi: within-group effect size for the intervention group; Wdt=within-group effect size for the delayed-treatment group.

^c^Estimated mean after completing treatment=12.43, observed mean after completing treatment=11.34.

**Table 3:** Proportion of participants reaching the criteria for clinically significant improvement on the Beck Depression Inventory-II (BDI-II) at 7 weeks of treatment and results of chi-square tests (χ^2^).

Treatment response	Full sample, n (%) (N=106)			Baseline BDI-II above clinical cutoff,^a^n (%) (n=90)			Current major depressive episode diagnosis, n (%) (n=51)		
	Intervention (n=52)	Control (n=54)	χ^2^_1_	Intervention (n=42)	Control (n=48)	χ^2^_1_	Intervention (n=26)	Control (n=25)	χ^2^_1_
Recovered	17 (32.7)	5 (9.3)	8.8^b^	15 (35.7)	5 (10.4)	8.3^b^	8 (30.8)	3 (12.0)	2.7
Improved	8 (15.4)	5 (9.3)	0.9	5 (11.9)	4 (8.3)	0.3	6 (23.1)	3 (12.0)	1.1
No change	26 (50.0)	41 (75.9)	7.7^b^	21 (50.0)	38 (79.2)	8.4^b^	11 (42.3)	18 (72.0)	4.6^c^
Deteriorated	1 (1.9)	3 (5.6)	0.3	1 (2.4)	1 (2.1)	0.01	1 (3.8)	1 (4.0)	0.001

^a^BDI-II>14.

^b^ *P*<.01.

^c^ *P*<.05.

### Therapist Effects

Therapist effects were investigated by looking at the interaction between therapist and time for the intervention group. The analyses indicated a significant difference between the 2 therapists when analyzing BDI-II scores up to 7 and 8 weeks of treatment (*P*=.03-.04). This effect no longer reached significance when including scores up to 10, 12, and 14 weeks of treatment (*P*=.05-.32). The analyses did not yield differential treatment effects across the 2 therapists for the HADS depression subscale (*P*=.87), nor for any other outcome measure (*P*=.50-.94). An exploratory linear regression analysis showed that symptom change in the intervention group was not significantly predicted by the total time spent by the therapists for any outcome measure (beta=–.12 to .26, *t*_*29-48*_=–0.81 to 1.52, *P*=.14-.96).

### Treatment Satisfaction

Table 4 shows the response frequencies for questions regarding satisfaction with the treatment. The results are reported for participants in both groups (intervention group: n=39; delayed-treatment control group: n=26) who completed the full treatment or parts of it. Overall satisfaction with the treatment was high, with 89% (58/65) giving the intervention as a whole a rating of 4 or 5 on a scale with 5 being very satisfied (see Table 4). Most participants also indicated that they would recommend the combined intervention to a friend with a similar problem. The ratings of the MoodGYM program were positive, but somewhat more moderate with between 50% and 60% giving clearly positive ratings (4 or 5 on the 5-point scales, see Table 4) to the benefit of the program, the usefulness of the exercises, and the relevance of the thematic content, and none rating the program as not useful or relevant. The benefit of the treatment sessions and the relationship with the therapist were rated positively by more than 90% (60/65 and 64/65, respectively) of the participants.

### Service Use and Work Status After Treatment

Of the 76 participants who completed the follow-up assessment, 45% (19/42) of participants in the intervention group and 38% (13/34) of participants in the control group had received treatment for mental health problems during the 6-month follow-up period. Two participants (3%) had been hospitalized, 19 (25%) had used antidepressant medication (15 currently using), 26 (34%) had received psychological treatment individually or group therapy, and 16 (21%) had received treatment from their GP. Of the 19 participants reporting use of antidepressants, only 6 had commenced this treatment during the follow-up period.

With regard to work status, 6 of 42 respondents (14%) in the intervention group reported that they had been on sick leave at some point in the follow-up period due to feeling tired, stressed, or experiencing mental health problems, whereas 9 of 34 respondents (26%) in the control group reported sick leave during this period.

**Table 4:** Response frequencies regarding satisfaction and experiences with the treatment (n=65).

Item		Satisfaction/experience scale				
		1	2	3	4	5
**Overall satisfaction with the treatment**						
	Scale	Very dissatisfied				Very satisfied
	n (%)	0 (0.0)	0 (0.0)	7 (10.8)	40 (61.5)	18 (27.7)
**Change in symptoms**						
	Scale	Much worse		Neither nor		Much improved
	n (%)	0 (0.0)	2 (3.1)	16 (24.6)	26 (40.0)	21 (32.3)
**Would recommend the treatment to a friend with a similar problem**						
	Scale	Definitely not				Yes, definitely
	n (%)	0 (0.0)	0 (0.0)	4 (6.2)	21 (32.3)	40 (61.5)
**Benefit of using MoodGYM**						
	Scale	No benefit				Highly beneficial
	n (%)	0 (0.0)	4 (6.2)	24 (36.9)	32 (49.2)	5 (7.7)
**The usefulness of the exercises in MoodGYM**						
	Scale	Not useful				Very useful
	n (%)	0 (0.0)	5 (7.7)	23 (35.4)	28 (43.1)	9 (13.8)
**Relevance of the thematic content of MoodGYM** ^**a**^						
	Scale	No relevance				Highly relevant
	n (%)	0 (0.0)	I: 4 (10.3) C: 1 (3.8)	I: 20 (51.3) C: 6 (23.1)	I: 10 (25.6) C: 13 (50.0)	I: 5 (12.8) C: 6 (23.1)
**Benefit of the follow-up sessions**						
	Scale	No benefit				Highly beneficial
	n (%)	0 (0.0)	0 (0.0)	5 (7.7)	34 (52.3)	26 (40.0)
**Satisfaction with the number of sessions**						
	Scale	Too few		Just enough		Too many
	n (%)	1 (1.5)	6 (9.2)	55 (84.6)	3 (4.6)	0 (0.0)
**Relationship to the therapist**						
	Scale	Very negative				Very positive
	n (%)	0 (0.0)	0 (0.0)	1 (1.5)	14 (21.5)	50 (76.9)

^a^ *P*<.05, frequencies reported separately for the intervention (I) and delayed-treatment control (C) groups.

## Discussion

### Principal Findings

The results of the present study indicate that a guided self-help intervention combining the MoodGYM program with face-to-face therapist support can be effective in reducing depressive symptoms for a sample of mildly to moderately depressed individuals recruited from primary care. The intervention also had significant positive effects on symptoms of anxious worry, and participants experienced significant improvements in global satisfaction with life. At 6-month follow-up, positive gains in terms of reduction of depressive and anxious symptoms were largely maintained, whereas improvements in life satisfaction were partly maintained. The rate of nonadherence (40%) was moderate and the evaluations of the treatment as a whole were predominately positive.

These findings are consistent with previous research in which favorable outcomes have been shown for treatments combining MoodGYM and face-to-face support from a professional [29,37]. The trials are not fully comparable, though, because the present study used a delayed-treatment control condition, whereas both previous trials used comparison groups that received more active treatments. This makes direct comparisons of between-group effect sizes difficult. However, the effect of guided self-help for mild to moderate depression using other Internet-based programs has generally been in the moderate to large range [19,27]. The magnitude of the between-group effect size on depression measured with BDI-II in this study was within this range, but somewhat below average, whereas the effects on SWLS and on depression and anxiety measured with HADS were commensurable to effects found previously for similar measures [12-14,27,99,100]. Also, the rates of recovery and of improvement and recovery combined (33% and 48%, respectively) are in good accordance with results of prior investigations, in which clinically significant improvement and recovery have varied between 25% and 50% [12,14,15,30,100]. The nature of the guidance provided in these studies is somewhat heterogeneous, with some studies, such as the present study, defining guidance as active engagement in the therapeutic process, whereas several other studies have focused primarily on providing feedback and encouragement. However, there is no indication of significantly differential treatment effects depending on the nature of the guidance to date [19,100]. The somewhat smaller between-group differences of the present study must be seen in relation to the relatively high degree of positive change in the control group, with effect sizes being in the small to moderate range for several measures (see Table 2). Almost half of the sample did not fulfill the criteria for a major depressive episode on enrollment in the trial, and previous studies of minor depression have shown high rates of placebo response in primary care patients [101] and substantial likelihood of spontaneous remission in the general population [102]. In the present study, the control group was also free to access usual treatment in general practice during the waiting period. In addition, prior to entering the study control participants participated in a screening session in which they had the opportunity to describe their problems, something that could have a therapeutic effect per se. These factors may partly explain the positive gains in the delayed-treatment group and, hence, the modest differences in outcome between the groups.

The results of the 6-month follow-up are also encouraging in that improvements of depression and anxiety symptoms were largely maintained, and the number of participants reporting sick leave due to mental health problems was substantially smaller during follow-up compared to baseline (20% and 53%, respectively). The proportion of participants using antidepressant medication at baseline and during the follow-up period was comparable, but there was a considerable increase in the number of participants who had accessed psychological treatment during follow-up (34%) compared to baseline (10%). This is, however, not surprising because many participants had already been referred to such treatment when entering the project, but may still have contributed to further improvements and maintenance of symptom reduction. For some measures, particularly the depression subscale of the HADS and the SWLS, there was a tendency toward an increase in symptoms and lowering of life satisfaction during the follow-up period. The inclusion of booster sessions after the completion of the active treatment phase could be a measure to accomplish continued use of helpful techniques and skills and the prevention of symptom relapse. Further research is needed to clarify this issue.

The results of the present trial are also consistent with research suggesting that MoodGYM, despite its main focus on depressive thought content, can have significant positive effects on anxiety symptoms [29,35,103]. In the present study, significant treatment effects were found for anxiety symptoms measured with HADS, but not with BAI. The questions on the HADS focus primarily on symptoms such as worry, nervousness, and not being able to relax. This is in good accordance with the core symptoms of the Goldberg Anxiety Scale [104], which has been used in several studies with MoodGYM. In comparison, the BAI incorporates both these subjective anxiety symptoms, as well as 3 more somatic symptom clusters: neurophysiological, autonomic, and panic [63]. The MoodGYM program focuses primarily on restructuring dysfunctional thinking and does not include an introduction to the physiology of anxiety or other treatment techniques for anxiety. Thus, the present results, with the program showing effects on anxious worry but not on more physiological symptoms, seem to be in-line with the thematic focus of the program.

The adherence rate in the present study is comparable to that observed in other online guided interventions, in which adherence varied between 55% and 75% [13,14,23,30,99]. The rates of adherence are also comparable to those seen in other psychotherapy research and in regular clinical practice [105-107]. This level of adherence in the current study, and the high proportion (89%) of completers reporting being satisfied with the treatment, points to the acceptability of the intervention. The evaluation of the MoodGYM program was somewhat more moderate with between 50% and 60% allocating an unambiguous positive rating to the benefit and relevance of the program. This moderate level of satisfaction in the present adult sample may arise from the fact that the MoodGYM program was originally targeted at youth and young adults. Although the therapists emphasized the applicability of the principles for all age groups when introducing the intervention, and many participants managed well to make use of the content, participants frequently characterized the program as “too young.” As these aspects of treatment were not formally measured, these factors require further investigation to be properly elucidated.

### Strengths and Limitations

The current study was designed to trial a treatment procedure prior to its evaluation in general practice. Therefore, we sought to ensure a high level of internal validity while at the same time aiming to increase external validity by reflecting the heterogeneity of patients in real clinical practice. One strength of the study is the relatively heterogeneous sample of participants with regard to the range of depression and anxiety symptoms. There was also substantial comorbidity with anxiety disorders, although lower than rates found in population-based studies [108,109]. The fact that 83% of screened participants were found eligible also indicates that the sample is representative of those who opted for this choice of treatment.

An overarching focus in designing the intervention was feasibility for implementation in general practice. Studies have suggested that GPs may find the implementation of CBT techniques too time-consuming [49,110]. Therefore, sessions were primarily supportive and structured by the Web-based program. To allow for flexibility and increase feasibility, a guideline script rather than a more comprehensive manual guided each consultation. This lack of rigid standardization may have introduced some variability in treatment fidelity.

Blinding of participants was not possible for obvious reasons in this trial. However, the control group was blinded to the status of the waitlist as a control condition. Waitlists for treatment is the norm in Norwegian mental health care, and the short wait for the present treatment compared to other treatment options, may have minimized negative effects (“nocebo effects”) in the control group.

The present study also has several limitations that need to be addressed. First, the design of the study with only 1 intervention group receiving a compound of intervention elements does not allow for tests of the specific contribution of MoodGYM and the face-to-face consultations. Second, the lack of allocation concealment and blinding, and the role of the first and second authors as therapists in the trial introduced a risk of bias that may have inflated the treatment effects. Unfortunately, resource constraints prevented the use of independent therapists. The use of self-report measures rather than therapist assessments does alleviate this problem to some degree. Biased outcome assessments were further prevented by ensuring that a research assistant without knowledge of the participants’ condition assignment collected posttest data. Third, the sole reliance on self-report is a limitation in itself. Independent preassessments and postassessments by a clinician blinded to condition allocation would have been preferable and would have strengthened the results. Fourth, at preintervention there was a lack of comparability in diagnosed anxiety between the groups, with a significantly larger proportion of the control group fulfilling the criteria for an anxiety disorder. Despite this difference in diagnosed anxiety, scores on 2 different anxiety scales (HADS and BAI) were not significantly different, which suggests comparable anxiety levels in the groups. To minimize the effect of differences in anxiety, all primary analyses were controlled for anxiety level as measured with BAI, for which the observed difference was most significant. Fifth, the use of an unequal number of assessments of depressive symptoms in the 2 groups, with the intervention group having weekly assessments and the control group only completing a pretest and posttest, may have resulted in more favorable effects in the intervention group because of measurement effects. Previous studies of nonclinical samples have indicated that scores on the BDI tend to decrease with repeated administration [111,112]. Whether this holds for clinical samples is less certain. In this study, the effect of repeated measurement cannot be clearly distinguished from the treatment effect. However, comparable effects were also found for symptoms assessed only preintervention and postintervention in both groups. This indicates that the treatment had beneficial effects over and above possible measurement effects. Still, in light of this limitation, the results must be interpreted with caution. Sixth, the use of different administration formats for the assessments of the treatment phase and follow-up, (paper-and-pencil vs online questionnaires, respectively) can potentially introduce measurement bias. Although the 2 formats correlate highly, a previous study reported a significant difference in mean scores on the BDI-II and BAI, which makes switching of formats problematic [59]. Despite this limitation, the results should not be considered weakened for most measures because the direction of differences has generally suggested that online versions tend to inflate estimations of symptom severity and lower ratings of quality of life [59,71,76], with the exception of BAI, for which Carlbring et al [59] found that means on the online version were lower compared to the paper-and-pencil version. The reliability of the 6-month follow-up results for the BAI may, therefore, be limited. Seventh, the multiplicity of outcomes increases the risk of type I errors. However, the main findings of the present trial would still be significant when employing the Bonferroni correction. This indicates the robustness of the findings. Finally, although the heterogeneity of the sample and the recruitment from primary care is a strength, the generalizability of the results is uncertain because the sample was a self-selected group. Based on the notifications by the GPs when informing a patient of the study, the estimated uptake (meeting up for screening) was 39% (128 of 325 who received information), which is slightly greater than the median uptake for computerized CBT [55]. Considering the extra barriers imposed by the research activities, this uptake rate is relatively high and indicates the possible acceptability of this treatment among depressed primary care patients. It also strengthens the generalizability of the results, by indicating that the self-selected group may be representative of a considerable proportion of the targeted group of primary care patients.

### Potential Clinical Implications and Further Research

The positive treatment effects found for the intervention in the present study are encouraging and suggest that this intervention may have a potential for use in a stepped-care approach. The demand for mental health treatment is higher than what can be met by the current number of trained clinicians [113]. To increase availability of treatment, beneficial interventions must be delivered as efficiently as possible to as many people as possible. The present intervention is time-limited, and because the CBT elements are largely delivered by the program, primary care therapists with some training in CBT and MoodGYM, should be able to provide adequate guidance. In fact, studies show that guidance may be delivered effectively not only by trained clinicians, but also by mental health workers with limited experience and by computer technicians [114,115]. Thus, dissemination of the current intervention to regular primary care could be a step toward increasing access to psychological therapies. However, the moderate ratings of the benefit and relevance of the content of the Web-based program by an adult population points to the need for a variety of Web-based tools to make such treatments acceptable for a wider audience.

For practical reasons, we chose to use psychologists for this first evaluation. Therefore, further research is needed to determine if the present intervention would be as effective and acceptable in regular clinical practice when delivered by GPs or other primary care therapists. It may also be noted that the present intervention was more time-intensive than most other guided self-help interventions. However, since the role of the clinician was mainly supportive and facilitative and the main therapeutic input was delivered through a standardized treatment package, the intervention was regarded conceptually as a guided self-help intervention [31]. Similar effects have been found for low- and high-intensity guided Internet-based psychotherapy [19]. Further research should investigate if the present intervention with more limited therapist support could yield similar effects.

### Conclusion

Despite its limitations, the present study indicates that an intervention combining the MoodGYM program with therapist support can be an effective treatment of depression in a sample of primary care patients. The intervention not only alleviates depressive symptoms, but also has positive and significant effects on symptoms of anxious worry and global satisfaction with life. Positive gains in terms of reduction of depressive and anxious symptoms were largely maintained at 6-month follow-up, and improvements in life satisfaction were partly maintained. Moderate rates of nonadherence and predominately positive evaluations of the treatment as a whole also indicates the acceptability of the intervention. The intervention was designed to be suitable for implementation in primary health care, and could have a potential for use in a stepped-care approach. However, further research is necessary to determine whether it is equally effective when delivered in regular primary health care and whether the inclusion of booster sessions could further improve symptom maintenance. Further research is also needed to investigate whether the intervention is truly acceptable for the wider group of primary care patients and whether it is considered feasible and acceptable by GPs or other primary care therapists.

## Multimedia Appendix 1

CONSORT-EHEALTH checklist V1.6.2 [53].

## Multimedia Appendix 2

Trial Protocol as requested in CONSORT-EHEALTH [53].

## Multimedia Appendix 3

Trial information to participants as requested in CONSORT-EHEALTH [53].

## Abbreviations

AUDIT: The Alcohol Use Disorder Identification Test
ANOVA: analysis of variance
ANCOVA: analysis of covariance
ANU: Australian National University
BAI: Beck Anxiety Inventory
BDI-II: Beck Depression Inventory, second edition
CBT: cognitive behavioral therapy
DSM-IV: Diagnostic and Statistical Manual of Mental Disorders, 4th edition
DUDIT: The Drug Use Disorder Identification Test
EQ-5D: EuroQol Group 5-Dimension Self-Report Questionnaire
EQ Index: index score of the EQ-5D
EQ VAS: visual analog scale of the EQ-5D
GP: general practitioner
HADS: Hospital Anxiety and Depression Scale
ICD-10: International Classification of Diseases, 10th edition
ITT: intention-to-treat
SWLS: Satisfaction with Life Scale
