# Supplementary material for: The Clinical Effectiveness of Web-Based Cognitive Behavioral Therapy With Face-to-Face Therapist Support for Depressed Primary Care Patients: Randomized Controlled Trial
Source: J Med Internet Res. 2013 Aug 5;15(8):e153. doi: 10.2196/jmir.2714 (PMC3742404; doi:10.2196/jmir.2714)
Supplement: Supplementary file 2 [file jmir_v15i8e153_app2.pdf]

Internet-based cognitive therapy combined with short follow-up consultations in the treatment of patients with depression:
A novel model for rapid psychological treatment with limited therapist contact.

Proposal for a joint project application from the Department of Community medicine (General Practice Research Unit) and the Department of Psychology at the University of Tromsø.

The project is aiming toward a more efficient treatment of patients with mild to moderate depression from general practitioners (GPs) and waitlists at district psychiatric centers. This will be accomplished through a randomized controlled trial on the use of an Internet-based cognitive behavior therapy program. The project will investigate if this novel approach of treatment can be used to improve the efficiency of psychologists and GPs in the treatment of mild to moderate depression.

1 Background

Mental disorders account for about 13% of the total disease burden in the world (WHO, 2008). In Norwegian general practice about one third of all consultations are due to mental health problems. One third of the population will experience a mental health problem during their lifetime. The prevalence of mental disorders is 9% at any time for women and 5% for men. Fifteen per cent of long-term sick leave (more than 8 weeks), and thirty per cent of all permanent disabilities in Norway are due to mental disorders. Ninety per cent of all psychological problems are treated in general practice (Hunskår, 2003).

In Australia mental disorders and substance misuse account for nearly 30% of all health-related disability (Whiteford, 2008). Depression is the leading cause of disability among all health conditions for both genders. General practitioners are the most common providers of mental health care. Seventy six per cent of patients who are treated for mental health problems are receiving their mental health care in general practice (Whiteford, 2008).

Also in Norway depression is a leading cause of disability, social and economic burden. The prevalence among females is more than double compared to males. According to Mental Health Norway (2004) almost 300 000 Norwegians suffer from depression, but only 50 000 seek help from public health services. General practitioners use depression as a diagnosis in about 5% of their consultations, whereas 3% are diagnosed with anxiety (Hunskår, 2003).

Depression: symptoms, vulnerability and treatments

Depression includes emotional (e.g. depressed mood), motivational (e.g. loss of interest or pleasure), cognitive (e.g. negative thoughts, feelings of hopelessness), and somatic (e.g. loss of energy, sleep disturbances) symptoms (APA, DSM IV, 1994). Almost everybody will experience some of these symptoms to some extent. However, when the symptoms become too many, too intense and long lasting, to such an extent that they interfere with social life, studies and working life, the depression is described as a major depressive episode that requires treatment.

In an epidemiological study from six European countries it was found that about 17% of the population reported some experience of depression during the last six months, whereas major depression was reported by 7% (Lepine, Gastpar, et al., 1997). These findings are comparable to rates reported in a Norwegian sample, in which the point prevalence of clinical depression was between 3% and 5%, and 24% of women and 10% of men reported that they had experienced a major depression during their lifetime (Kringlen et al., 2001).

Accordingly, findings clearly show the importance of a prevention focus in the treatment of depression. Strikingly, little research has been devoted to the prevention of common mental illness. Effective programs to prevent the development of depressive symptoms into clinical depression that require professional treatment, will potentially reduce human suffering, and may also have substantial economical benefits (McCrone, Knapp, Proudfoot, et al., 2004).

According to Beck's cognitive theory of depression, negative thoughts play a central role in depression. The aim of cognitive behavior therapy (CBT) is to change negative and dysfunctional patterns of thinking in vulnerable individuals to prevent depressive symptoms to develop. This will in turn facilitate positive coping skills when faced with stressful situations (Beck, 1967; 1976; Beck et al., 1979). Several studies have documented that CBT is effective in treating mild to moderate depression, and that CBT can be

as effective as antidepressant medication. CBT can be structured and manualized, making it suitable for self-help procedures (Churchill, Hunot, et al., 2001; Andersson et al. 2005; Mackinnon et al., 2008).

Reluctance to help seeking amongst depressed requires new approaches to treatment

The major obstacles in help seeking for depression are the depressive symptoms themselves, the knowledge base of the individual, and geographical distance. Estimates indicate that more than half of patients with major depression do not consult a health care professional (Mental Health Norway, 2004). Similar findings were also obtained in another Norwegian study, in which one third of the participants had felt a need for psychological help, and as many as two thirds of these refrained from seeking help. Some key predictors of reluctance to seek help in this sample were young age and depressive symptoms (Skarsvåg, 2004).

Several researchers have investigated why people avoid seeking help for psychological problems. Fear of stigma and of other negative consequences of seeking help, negative evaluation of the helper and the wish to solve the problem themselves have been indicated as the most important factors (Priest et al., 1996; Jorm et al., 1997; Johannesen, 1998; Hillert, Sandmann et al., 1999; Wrigley et al., 2005; Barney, Griffiths et al. 2006). Furthermore, depression itself has been established as an avoidance factor in help seeking.

Cognitive therapy-based interventions provided by GPs represent a potential resource to reduce the costs of depression and improve outcomes (Whiteford, 2008). The traditional specialized psychiatric health care system may not fit everyone. For example, the need for anonymity, spontaneous behavior, especially in young people, and geographical distance, may be important reasons necessitating alternative health promotion programs. Mental health services are generally recognized as being less available in rural and remote areas (Caldwell et al., 2004). In the Northern Norway Health Region where more than 32% of the citizens live in rural areas, there is 0.59 psychologist per 1000 inhabitants compared to 1.11 per 1000 in the metropolitan area (Oslo and Akershus) (Statistisk sentralbyrå, 2009). This adds a heavy burden to the providers of mental health services in the northern region, which is characterized by long travelling distances to regional hospitals, longer waitlists, and fewer specialized services and health care professionals in the rural areas than in the cities (Helse Nord, 2005). When patients finally contact the GP for help they will normally have to wait 3-6 months for treatment in the specialized mental health service. To overcome both the patient delay and the referral delay there is a need for new approaches to primary treatment of patients, and for a more efficient use of the existing limited resources in the specialized health care system.

Internet as a potent medium to deliver health promotion programs

Several studies indicate that patients generally prefer psychological therapies to medication. Thus, computerized therapy programs could be valuable tools for GPs in managing patients with mental health problems (Proudfoot et al., 2003, Cavanagh, Shapiro et al., 2006; Shandley et al., 2008). Computerized CBT has since 1999 been successfully used in Great Britain as a psychological treatment for common mental health problems presented in primary care. Economical analyses have shown that computer-delivered CBT is cost-effective, and have revealed a highly competitive cost per quality-adjusted life year (McCrone, Knapp, Proudfoot et al., 2004).

Research on mental health interventions by means of the Internet has shown that allowing people anonymity, and freeing them from the limitations of time and geographical space, result in beneficial effects (McCrone, Knapp et al., 2004; Proudfoot et al., 2004).

Internet-based interventions are easy to keep up to date (Oenema et al, 2001), and can be managed by the intervention editor group. Evaluation of such interventions may also be more flexible, faster, more comprehensive and more cost-effective. Whereas traditional mass-media interventions have reached large portions of the population at a relatively low cost, they have proven less effective than individual interventions. Internet provides the opportunity of combining these strategies at an even lower cost by utilizing computerized tailoring (Kreuter & Skinner, 2000).

Undoubtedly, Internet-based interventions represent a potential option for bridging service gaps with regard to the availability of specialized services to people experiencing geographic and mobility constraints, such as people living in rural and under-served communities, and people with disabilities (Conrad, 1998). Internet based counseling can be used as an adjunct to traditional face-to-face therapy, as a substitute for face-to-face contact with a mental health professional, or it can be the only way for some people who would never consider to see a therapist face-to-face (Grochol, 2001; Berger et al. 2009).

In collaboration with Australian National University (ANU) in Canberra, a Norwegian version combining both BluePages and MoodGYM in a "BlueMood" program has been translated and adapted at the Department of Psychology, University of Tromsø in October 2005.

The efficacy of using cognitive therapy in general practice

The efficacy of CBT in general practice has already been demonstrated in numerous papers within the last couple of years. Such treatments achieve comparable patient outcomes as treatments delivered by psychologists. Cognitive therapy may prove useful in the treatment of several disorders. Very encouraging results have been found for a combination of internet-based and general practitioner-supported therapy in the treatment of panic disorders (Shandley et al., 2008). In another Australian study the cost effectiveness of a CBT-based self-help program in conjunction with GP-treatment has been shown. Somatoform disorders can be treated by early intervention in general practice if the GP is properly trained (Forbes et al., 2007). General practitioners have an important role to play in helping patients after exposure to severe psychological trauma (Forbes et al., 2007). Cognitive therapy offers good models for treating posttraumatic stress reactions, which can easily be implemented in general practice (Mørch & Rosenberg, 2005). The sustainability of such therapies would depend on funding, workforce availability and acceptability by both patients and providers (Mihalopulos et al., 2005).

In a recent supplement of the Medical Journal of Australia, Whiteford (2008) wrote: “A longstanding concern has been whether GPs are able to detect common mental disorders in their patients”. Clarke et al. (2008) examined the way distress and depression is perceived by GPs, and developed a taxonomy that may be useful in general practice settings. Wilhelm et al. (2008) have discussed the value of screening tools to increase recognition of patients with mental disorders. Given that the requirement for mandatory training (present in the Better Outcomes in Mental Health Care program) was removed in the Better Access program, the issue of training GPs is critical. Blashki et al. (2008) demonstrated that competency in cognitive behavioral therapy, at least in highly motivated GPs, can be improved with relatively brief training sessions. In Denmark, the need for using CBT in general practice has been the topic of a series of more than 10 articles in Månedsskrift for Praktisk Lægegerning. The editor of these articles Marianne Rosendal (2007) wrote as follows: “Many of these (cognitive techniques) are easily accessible and can be implemented in the clinical day to day work in general practice”.

Interestingly, in Norway there has been little coordinated effort to introduce CBT into general practice in spite of an active interest from personnel both in the secondary health sector and among nursing personnel. Recently, however, Norwegian GPs are beginning to show an interest in CBT (Johannessen, 2006; Kolstrup, 2008).

The GP’s role in treatment and referral of patients with mental health problems

When patients seek help for their psychological problems they seek the advice and help of a GP. If the treatment they received immediately after the first contact could be as beneficial as possible, the risk that relatively trivial psychological problems develop into more serious psychological and social problems while waiting for treatment, would diminish.

Australian findings show that patients generally prefer psychological therapies to medication, and, if possible, that the treatment is carried out in general practice. Therefore, the main focus of activity aimed at reducing the burden of common mental disorders in Australia is on primary care. Specialist mental health services play a supporting, but not central role (Whiteford, 2008).

Since CBT is now well documented as a superior way of treating a variety of mental health problems, the government of the United Kingdom has decided to implement this treatment in the majority of the new health care centers that are being established as the basis of the National Health System (NHS).

David (2006) argues that CBT is difficult to apply in general practice in United Kingdom due to the constraints of short consultations, but that a general knowledge of the ideas behind CBT is very useful to all GPs as part of their consultation skills. However, as pointed out by David (2006) and King et al. (2000), there is a need for CBT to be modified to suit general practice. These modifications include simplified modes of delivery, optimal ways of combining medications with CBT, and minimizing the risk of recurrence after the cessation of treatment (Beattie et al., 2009). In addition, the treatment needs modifications in line with the educational and administrative framework of the specific country.

In many Norwegian primary health care clinics there is already an established and well-functioning cooperation between psychologists and GPs in the treatment of patients. This is due to the decentralized structure of psychiatry in Norway. In some areas “acute teams” from regional psychiatric centers have cooperated extremely well with local GPs. The combined use of the expertise of the GPs and the members of the acute teams has proven invaluable in solving complex psychiatric problems. Furthermore, this approach has often resulted in an invaluable mutual learning effect. If this tradition of good cooperation could be used more efficiently as a basis for treatment and education, many patients could be treated outside the specialized health service. Furthermore, there are already good economic and educational incentives in place to motivate GPs for this type of cooperation.

The already existing organizational structures in Norwegian general practice with both economic and educational incentives for a close cooperation between psychologists/psychiatrists and GPs should facilitate the implementation of CBT in Norwegian general practice, and thus ensure an improved and faster treatment of patients, as well as result in a reduced use of the already scarce resources in the specialized health care system.

Unfortunately, a set of structural and mental barriers seems to hamper the more widespread use of CBT in Norwegian general practice (Kolstrup, 2007; 2008). Consequently, there is a need for more knowledge about the experiences from other countries and about the practical and psychological mechanisms impeding the implementation of CBT in Norwegian general practice.

2 General objectives

There are insufficient psychologists to provide psychological treatments for all adults exhibiting depression in general practice settings. Moreover, general practitioners are typically time-poor and are not trained to deliver psychological therapy. The current project aims to trial a procedure for more efficient treatment of mild to moderate depression in general practice. The study will involve a randomized controlled trial using an Internet-based cognitive behavior therapy program (MoodGYM) combined with brief follow-up consultations with a health professional (psychologist/general practitioner), and tailored e-mails between treatment sessions. The project is designed to determine if this novel approach can improve the efficiency of psychologists and general practitioners in the treatment of mild to moderate depression. In addition, the project will allow us to investigate the effect of MoodGYM in a clinical sample.

Basic research questions

The primary aim is to explore the feasibility, efficacy and the patients' experience with and acceptability of an Internet-based self-help program combined with brief face-to-face treatment and tailored e-mails between treatment-sessions, for adults with depression attending an outpatient general practice clinic. In addition, the project aims to identify the optimal screening tool for identifying depression in general practice by comparing different screening tools, and to explore the development of a clinician's manual that can be used both by general practitioners and psychologists

The primary hypothesis is that the Internet-based program will lead to a larger reduction in depressive symptoms than a waitlist control condition. The study will also:

- Examine how the level of depressive symptoms is related to the use of the Internet-based intervention program;
- Investigate how age, gender and educational background are related to the use of the Internet-based intervention program;
- Determine the attrition rate and explore reasons for drop-out from the intervention;
- Examine how patients' locus of control, self-efficacy and self-esteem are related to the use of the intervention;
- Investigate how the patients' attitudes and expectations toward the treatment, and intention of use are related to the use of MoodGYM;
- Investigate patient satisfaction with the treatment program.

A qualitative enquiry aims to:

- Identify and describe the changes that have happened in the patients' life during the time they received treatment;
- Identify and describe what motivates patients to progress in treatment;
- Critically examine the treatment model as experienced by the patient.

Feasibility

There are potential difficulties with this project: The recruitment of patients, and the willingness and ability of patients to adapt to 20-minute consultations, and the use of new technology in terms of an Internet-based therapy program. We have tried to minimize these problems by:

1. Recruitment of patients from waitlists at Silsand DPS.
2. The project manager is a practicing GP with an extensive network of colleagues in general practice.
3. The follow-up of patients will be concentrated to weekly consultations, thereby ensuring that the motivation of the patients is kept up.
4. Informing the patients that the consultations are different from regular 45-minute consultations with a psychologist.
5. By including only patients with access to the Internet at home, we can assume that they are familiar with the use of this technology.

Methods

This study will comprise a 6-week randomized controlled trial in Norway conducted in an outpatient clinic.

Participants and a priori sample size estimation

Previous studies of Internet-based self-help for depression and anxiety have shown effect sizes in the small to large range (Sampson, Kolodinsky, & Greeno, 1997; Andersson et al., 2005). We estimate effect sizes of 0.2 for the control group and 0.6 for the BlueMood intervention. To detect differences of this size between two groups at a .05 significance level and with .80 power, a sample of 50 participants per condition is required. To allow for an average attrition rate of 10%, a total of 55 participants will be required in each group.

Individuals aged 18 to 65 years with mild to moderate symptoms of depression will be recruited through 12 GP-practices in Tromsø and waitlists at Silsand DPS. Participation will be voluntary and based on informed consent. Participants will be informed that they can withdraw at any time without explanation. Patients with symptoms of depression will be informed of the study by their GP. All potentially eligible participants will be screened for inclusion into the study by a general practitioner or a psychologist. The inclusion criteria will be (i) mild to moderate symptoms of depression as indicated by a score between 10 and 40 on Beck Depression Inventory-II (BDI-II); and (ii) access to the Internet between follow-up sessions. Participants will be excluded from the trial if (i) they receive a score of 17 or more on the suicidal ideation module in the Mini-International Neuropsychiatric Interview (MINI), in order to minimize the risk of including participants in need of more extensive treatment; (ii) current alcohol use disorder as indicated by a score above 20 on the Alcohol Use Disorder Identification Test. This cutoff score was chosen to exclude patients who were clearly in need of further diagnostic evaluation for alcohol dependence. Patients with scores between 16 and 19 were included in the study, but alcohol use was monitored during treatment; (iii) current drug use disorder as indicated by scores above 25 on the Drug Use Disorder Identification Test; (iv) current psychosis (screened by means of the MINI); (v) individuals currently receiving CBT from a psychologist or psychiatrist. Individuals who use antidepressant medication will be stabilized for 1 month prior to evaluation of diagnostic eligibility.

The intervention

The intervention will be based on the Norwegian version of MoodGYM which is an Internet-based cognitive behavioral self-help program originally developed at the Australian National University. MoodGYM consists of interactive webpages used to deliver CBT. The site includes 5 cognitive behavioral training modules, and a personal workbook. Module 1 introduces the "characters" who model patterns of dysfunctional thinking, and demonstrates the way in which mood is influenced by thinking. Module 2 describes types of dysfunctional thinking, and the methods to overcome them. Module 3 provides behavioral methods to overcome dysfunctional thinking, and includes sections on assertiveness and self-esteem training. Module 4 assesses life-event stress and teaches relaxation techniques. Module 5 covers simple problem solving and typical responses to broken relationships.

Consenting participants will be randomized to receive either the MoodGYM program combined with consultations with a GP or psychologist or to a waitlist control condition. Participants allocated to the intervention arm of the study will be provided with a brief introduction to the MoodGYM program by a GP or psychologist. The participants will meet the same GP or psychologist for the screening, the introduction of the program and the treatment sessions. During the 6-week trial, the participants will be asked to work through the MoodGYM program (1 module each week), and attend a 20-minute follow up session with a psychologist/GP each week. They will also receive tailored e-mails between sessions aiming to increase treatment motivation.

During the 20-minute follow-up sessions, focus will be on the main theme of the last week's

MoodGYM module, and the patient's experiences of working with this theme. Symptoms of depression and anxiety will be monitored at every consultation, and changes in symptom levels are discussed. Therapists will also focus on motivating the patients to adhere to the treatment plan. The therapists follow the same plan for every consultation. This is not a manual to be adhered to strictly, but rather an outline describing important aspects for discussion with the patient, including important topics from every module.

The control group will consist of participants randomized to a waitlist for 6 weeks. After these 6 weeks the control group will receive the intervention described above.

All consenting participants will undergo a pretreatment, posttreatment and a 6-month posttreatment assessment concerning symptoms of anxiety and depression. Before entering treatment the control group will also complete a questionnaire, which is identical to the intervention group's posttreatment assessment.

Measures

Table 2 gives an overview over the measures used during different phases of the project. The measures used in this project are described in more detail with references in Appendix B.

Table 2. Measures used in different phases in the project.

Measures	Pretreatment measures	Online assessments	Posttreatment measures
Descriptive variables/socio-demographic variables	X		
MINI Neuropsychiatric Interview	X		
MINI Screen Questionnaire	X		
Beck Depression Inventory-II (BDI-II)	X		X
Beck Anxiety Inventory (BAI)	X		X
Goldberg Depression Scale		X	
Warpy Thoughts Quiz		X	
Hospital Anxiety and depression scale (HADS)	X		X
The Alcohol Use Identification Test	X		
The Drug Use Identification Test	X		
The Rosenberg Self Esteem Scale	X		
General Self-Efficacy Scale	X		
The Locus of control Scale	X		
Attitudes towards the treatment	X		
Satisfaction with treatment			X

Qualitative investigation of patients' experiences with the treatment program

This is a qualitative investigation with a phenomenological-hermeneutical approach inspired by the philosophy of Ricoeur (Ricoeur, 1976; Lindseth & Nordberg, 2004). One to one semi structured interviews of 45 to 60 minutes will be conducted to obtain empirical information about the patient's experience. The patient will not be interviewed by their therapist. When conducting the interview the patient will be encouraged to narrate, as freely as possible, about his/her experience with the topic chosen by the interviewer and by himself/herself.

To obtain information about changes in the patient's life he/she will be encouraged to describe experiences of activating social relations and possible changes/new dimensions in his/her life and everyday living. To explore the impact of the treatment the patient will be encouraged to elaborate about a certain situation he/she handled differently as a result of treatment. Further, it will be explored if the patient would describe himself/herself differently today than before treatment.

To explore motivation as a phenomenon the patient is encouraged to tell about what made him/her take part in this project. By mapping out what were important aspects of the treatment information and the treatment itself, motivation to start and/or to progress hopefully will be gained.

To examine how the patient experienced the treatment model he/she is invited to describe the treatment, and to evaluate the interactive model.

For a more extensive description of the questions on each topic, see enclosed interview guide.

3 Ethical Considerations

There are ethical issues involved in any study of individuals. The project has been designed to minimize harm to participants. It not only employs a self-help program, which has been successfully delivered to a large number of individuals, the participants also receive weekly treatment from a health professional. The instruments used at baseline and follow-up are standardized and internationally used. There are no known reports that they are causing harm among respondents. The voluntary basis of participation will also minimize the risk for the patient. The control group will receive the intervention after 6 weeks on a waitlist. Participants who during the trial experience a substantial worsening of symptoms will be offered more extensive follow-up or referral to specialized mental health care. All online interactions will adhere to the Norwegian Data Inspectorate's data security regulations regarding encryption, authentication and storage.

4 Dissemination of results

The proposed study is expected to be part of the basis of three PhD-theses, one for a medical doctor and two for clinical psychologists.

The general topics of the project will be investigated by three PhD-students. Results on the specific aims of the project will form the basis for a minimum of 3 articles, which will be submitted to international peer-reviewed journals. Furthermore, it will be encouraged to publish articles in Norwegian journals and teach at courses, and give papers and posters at conferences to disseminate the results to relevant groups of professionals in Norway. It will also be important to attend meetings for students and patient organizations. An educational course for GPs be disseminated through the Norsk Elektronisk Legehåndbok (NEL) ("Norwegian electronic handbook for doctors"; Johanessen, personal communication). This is presently the single most used source of information for Norwegian general practitioners, and is being introduced in Denmark, as well.

The target groups for the results of the study will be general practitioners, clinical psychologists, psychiatrists and health authorities, but also students within health care, patients and the community in general. It will also be important to reach groups of individuals who are especially vulnerable for developing depression, and who usually do not consult a health professional. Therefore, the findings from this project will be published and referred to within the context of popular science, like daily newspapers, magazines, radio and television.

Table 3 Tentative articles

**Tentative articles**
Internet-based cognitive therapy combined with short follow-up consultations in the treatment of patients with depression.
A comparative study of screening tools for depression in general practice.
How are patients' attitudes, expectations and intentions concerning treatment related to the use of an Internet-based intervention program for depression?
Qualitative investigation of patients' experiences of the treatment program.

5 Project timetable

Table 4 Main activities and milestones in the project period (year and quarter)

Milestones throughout the project	From		To	
Regional Ethics Committee	2009	4	2010	2
Patient recruitment	2010	3	2012	2
Data collection	2010	3	2012	2
Data Analysis	2012	2	2013	2
Articles	2012	2	2016	4

6 Research environment and project organization

The project is part of an international research collaboration between the Research Unit for General Practice and the Department of psychology (IPS) at the University of Tromsø, Silsand DPS, the Norwegian Institute of Public Health (NIPH) and the Australian National University (ANU).

University of Tromsø (UiT)
Research Unit for General Practice, Department of Community Medicine (ISM)
Associate professor Nils Kolstrup, PhD, MSc, MD is a specialist in general medicine and public health. He is currently working part time as a GP, and part time at the Department of Community Medicine at the University of Tromsø. He is affiliated to the Research Unit for General Medicine. He has extensive experience in supervising students. Kolstrup is a former member of the specialist committee for general medicine, a certified cognitive therapist, and associate leader of the "referansegruppe for kognitiv terapi". He has been an author of a book on CBT (Kolstrup, 2002), and several articles on CBT (Kolstrup, 2007; 2008).
Kolstrup will function in a supervisory role for the PhD-candidates.

Department of Psychology (IPS)
Professor Martin Eisemann, PhD, has published widely on depression and alcohol abuse and has a substantial experience in supervising doctoral candidates. Professor Knut Waterloo, PhD, and head of clinical services, is a certified specialist in clinical adult psychology and in clinical neuropsychology. Ove K. Lintvedt is a psychologist and computer engineer. He has been working with the BlueMood-project since 2005. Eisemann and Waterloo will function in supervisory roles for the PhD-candidates. The project will be co-located at the Department of Community Medicine (General Practice Research Unit) and the Department of Psychology at the University of Tromsø.

Norwegian Institute of Public Health (NIPH), Department of Mental Health
Arne Holte is director of the Department of Mental Health at NIPH and Professor in health psychology at the University of Oslo. His current focus is particularly on preventive health interventions, for which he advises The Norwegian health authorities in formulating national public policies. Holte will have a supervisory role for the PhD-candidates. In the last evaluation of the Norwegian research communities conducted by the Research Council of Norway, the Division of Epidemiology (which was then included in the Department of Mental Health) received the highest score ("excellent").

Australian National University (ANU)
Associate Professor Kathleen M. Griffiths, PhD, and Professor Helen Christensen developed the BluePages and MoodGYM. They have established the services at ANU's e-hub, which develops and evaluates Websites that deliver psychoeducation, psychological interventions, and clinical and consumer networking. ANU is eager to collaborate with Norwegian colleagues both from comparative perspectives, but also in finding innovative approaches to overcoming shared geographic barriers to public access to mental health support.

References

American Psychiatric Association. (1994). Diagnostic and statistical manual of mental disorders (4th ed.),. Washington, DC: Author.

Andersson, G., Bergström, J., Holländare, F., Carlbring, P., Kaldo, V., & Ekselius, L. (2005). Internet-based self-help for depression: randomised controlled trial. Br J Psychiatry. 187:456-61.

Austin, S.F., Sumbundu, A.D., Lykke, J., & Oestrich, I.H. (2008). Treating panic symptoms within everyday clinical settings: the feasibility of a group cognitive-behavioural intervention. Nord J Psychiatry. 14:1-7.

Babor, T. F., Higgins-Biddle, J. C., Saunders, J. B., & Monteiro, M. G. (2001). The Alcohol Use Disorders Identification Test: Guidelines for use in primary care Retrieved June 22, 2011, from http://whqlibdoc.who.int/hq/2001/who_msd_msb_01.6a.pdf

Barney, L.J., Griffiths, K.M., Jorm, A.F., & Christensen, H. (2006). Stigma about depression and its impact on help-seeking intentions. Australian And New Zealand Journal Of Psychiatry 2006, 40 - 51.

Beattie A, Shaw A, Kaur S, Kessler D.(2009) Primary-care patients' expectations and experiences of online cognitive behavioural therapy for depression: a qualitative study. Health Expect. 12(1):45-59

Beck, A.T. (1967). Depression: Clinical, experimental, and theoretical aspects. New York: Harper & Row.
Beck, AT. (1976). Cognitive therapy and the emotional disorders. New York: International Universities Press.
Beck, A.T., Rush, A.J., Shaw, B.F., & Emery, G. (1979). Cognitive therapy of depression. New York: Guilford Press.
Berger T, Hohl E & Caspar F.J Clin Psychol. (2009) Internet-based treatment for social phobia: a randomized controlled trial.May 12.]
Blashki,, G.A., Piterman, L., Meadows, G.N., et al. (2008). Impact of an educational intervention on general practitioners' skills in cognitive behavioural strategies: a randomised controlled trial. Med J Aust; 188,129-132.
Caldwell, T.M., Jorm, A.F. & Dear, K.B.G. (2004). Suicide and mental health in rural, remote and metropolitan areas in Australia. Medical Journal of Australia, 181, 10-14.
Cavanagh, K., Shapiro, D.A., Van Den Berg, S., Swain, S., Barkham, M., & Proudfoot J. (2006). The effectiveness of computerized cognitive behavioural therapy in routine care. British Journal of Clinical Psychology, 45, 499-514.
Churchill, R., Hunot, V., Corney, R., Knapp, M., McGuire, H., Tylee, A., & Wessely, S. (2001). A systematic review of controlled trials of the effectiveness of brief psychological treatments for depression. Health Technology Assessment, 5,35.
Conrad, K. (1998). Making telehealth a viable component of our national health care system. Professional psychology. Research and Practice, 29, 525–526.
Clarke, D.M., Cook, K., Smith, G.C., & Piterman, L. (2008). What do general practitioners think depression is? A taxonomy of distress and depression for general practice. Med J Aust; 188:12.
David, L. (2006). Using CBT in General Practice. The 10 minute Consultation. Bloxham; Scion Publishing Ltd.
Edelman, S. & Blashki G. (2007) Managing anxious patients: cognitive behaviour therapy in general practice. Aust Fam Physician. 36(4):212-4; 217-20.
Escobar, J.I., Gara, M.A., Diaz-Martinez, A.M., Interian, A., Warman, M., Allen, L.A., et al. (2007) Effectiveness of a time-limited cognitive behaviour therapy type intervention among primary care patients with medically unexplained symptoms. Ann Fam Med. 5(4):328-35
Forbes, D., Creamer, M.C., Phelps, A.J., Couineau, A.L., Cooper, J.A., Bryant, R.A.(2007) Treating adults with acute stress disorder and post-traumatic stress disorder in general practice: a clinical update. Med J Aust. 187(2):120-3
Grohol, J.M. (2001). Best practices of e-therapy: clarifying the definition of e-therapy,. Retrieved mai 15 2008 from http://www.psychcentral.com/best/best5.htm
Haavet, O.R., Christensen, K.S., Sirpal, M.K., & Haugen, W. (2007). Diagnosis of depression among adolescents--a clinical validation study of key questions and questionnaire. BMC Fam Pract. 13;8:41.
Helse Nord. (2005). Desentralisering av spesialisthelsetjenester i Helse Nord; 2005, retrived May 15 2008 from http://www.helse-nord.no/getfile.php/
Hillert, A., Sandmann, J., Ehmig, SC., Sobota, K., et al. (1999). The general public's cognitive and emotional perception of mental illness:an alternativ to attitude-research. In The Image of Madness: The Public Facing Mental Illness and Psychiatric Treatment. Edited by: Guimon J, Fischer W, Sartorius N. Basel: Karger; 56-71.
Johannessen, J. (1998). Early intervention and prevention in schizophrenia - experiences from a study in Stavanger, Norway. Psychiatria et Neurologia Japonica, 100, 511-522.
Johannessen, L.B. (2006). Vil tilby allmennleger kurs i kognitiv terapi. Tidsskr Nor Lægeforen. 126(19):2571.
Jorm, A.F., Korten, A.E., Jacomb, P.A., Christensen, H., Rodgers, B., & Pollitt P. (1997). "Mental health literacy": a survey of the public's ability to recognise mental disorders and their beliefs about the effectiveness of treatment. Medical Journal of Australia, 66, 182–186.
King, M., Sibbald, B., Ward, E., Bower, P., Lloyd, M., Gabbay, M., & Byford, S. (2000). Randomised controlled trial of non-directive counselling, cognitive-behaviour therapy and usual general practitioner care in the management of depression as well as mixed anxiety and depression in primary care. Health Technol Assess. 4(19):1-83.
Kolstrup, N. (2007). Nyttige grep i allmennmedisin. Dagens Medisin 18. oktober 2007:17.
Kolstrup, N. (2008). Hva er kognitiv terapi - og kan vi bruke det i allmennpraksis? (What is cognitive therapy - and can we use it in family practice?), Utposten; 37(1):7-13
Kreuter, M.W., & Skinner, C.S. (2000). Tailoring: what's in a name? Health Education Research, 15 (1), 1- 4.
Kringlen, E., Torgersen, S., & Cramer V.A. (2001). Norwegian psychiatric epidemiological study. Archives of General Psychiatry, 158, 1091-1098.

Lindseth, A. & Nordberg, A. (2004). A phenomenological hermeneutical method for researching lived experience. Scandinavian Journal of Caring Sciences, 18, 145-153.

Lepine, J.P., Gastpar, M., Mendlewicz, & J., Tylee, A. . (1997). Depression in the community: The first pan-European study DEPRES. International Clinical Psychopharmacology, 12, 19-29.

Mackinnon, A., Griffiths, K.M., & Christensen, H. (2008). Comparative randomised trial of online cognitive-behavioural therapy and an information website for depression: 12-month outcomes. Br J Psychiatry. 192(2):130-4.

McCrone, P., Knapp, M., Proudfoot, J. et al., (2004). Cost-effectiveness of computerised cognitive–behavioural therapy for anxiety and depression in primary care: randomised controlled trial. The British Journal of Psychiatry , 185, 55-62.

Mental Health Norway. Den nye folkesykdommen, 2004. Retrieved May 19, 2008, from http://www.mentalhelse.no/?module=Articles;action=Article.publicOpen;ID=4046

Mihalopoulos, C., Kiropoulos, L., Shih, S.T., Gunn, J., Blashki, G., & Meadows., G. (2005). Exploratory economic analyses of two primary care mental health projects: implications for sustainability. Med J Aust. 183(10 Suppl):73-6.

Mørch, M.M. & Rosenberg, N.K. eds. (2005). Kognitiv terapi. Modeller og metoder. København: Hans Reitzels forlag.

Oenema, A., Brug, J., & Lechner, L. (2001). Web-based tailored nutrition education: results of a randomized controlled trial. Health Education Research, 16 (6), 647-660.

Oestrich, I.H. & Kolstrup, N. (2002). Kognitiv terapi i lægepraksis En manual for læger. København: Dansk psykologisk forlag.

Oyama, O., Paltoo, C., & Greengold, J. (2007). Somatoform disorders. Am, ;76(9):1333-8.

Paperny, D., & Hedberg, V.A. (1999). Computer-Assisted Health Counselor Visits: A Low-Cost Model for Comprehensive Adolescent Preventive Services. Archive Paediatric Adolescence Medicine. V153(1), 63-67.

Priest, R.G., Vize, C., Roberts, A., Roberts, M., & Tylee, A. (1996). Lav people's attitudes to treatment for depression: results of opinion poll for Defeat Depression Campaign just before its launch. British Medical Journal, 313, 858–859.

Proudfoot, J., Goldberg, D., Mann, A., Everitt, B., Marks, I., & Gray, J.A. (2003). Computerized, interactive, multimedia cognitive-behavioural program for anxiety and depression in general practice. Psychological Medicine, 33(2), p. 217-227. Retrieved 15 may 2008 from http://www.biomedcentral.com/1471-2458/6/34

Ricoeur, P. (1976). Interpretation theory: Discourse and the Surplus of Meaning. Fort Worth, Tex.: The Texas Christian University Press.

Rosendal, M. (2007). Kognitiv adfærdsterapi i almen praksis. Månedsskrift for praktisk lægegerning 85:499-499

Sampson, J., Kolodinsky, R., & Greeno, B. Counseling on the information highway: future possibilities and potential problems. Journal of Counseling and Development, 1997;75 (3), 203-212.

Shandley, K., Austin. W., Klein, B., Pier, C., et al. (2008). Therapist-Assisted, Internet-Based Treatment for Panic Disorder: Can General Practitioners Achieve Comparable Patient Outcomes to Psychologists? Journal of Medical Internet Research, 10:e14. Retrieved from http://www.jmir.org/2008/2/e14

Starcevic, V. (2008). Treatment of panic disorder: recent developments and current status. Expert Rev Neurother. 8(8):1219-32

Skarsvåg, H.A. (2004). Seeking treatment or not? A study on mental helpseeking and its relation to needs, symptoms, person characteristica, experiences and attitudes in a student population. Unpublished main thesis for the Cand. psychol. Degree at the University of Tromsø.

Statistisk sentralbyrå. (2009). Retrieved from http://statbank.ssb.no/statistikkbanken/

Whiteford, H.A. (2008). Depression in primary care: expanding the evidence base for diagnosis and treatment. MJA, 188:101-102

WHO. (2008). Retrieved May 15 2008 from http://www.who.int/healthinfo/statistics/gbdwhoregiondaly2002.xls

Wilhelm, K.A., Finch, A.W., Davenport, T.A., & Hickie, I.B. (2008). What can alert the general practitioner to people whose common mental health problems are unrecognised? Med J Aust; 188: 114-118.

Wrigley, S., Jackson, H., Judd, F., & Komiti, A. (2005). The role of stigma and attitudes towards help seeking for mental health problems in a rural town. Australian & New Zealand Journal of Psychiatry, 39, 514-521

Zachrisson, H., Rödje, K., & Mykletun, A. (2006). Utilization of health services in relation to mental health problems in adolescents: A population based survey, BMC Public Health.
