# Supplementary material for: The Clinical Effectiveness of Web-Based Cognitive Behavioral Therapy With Face-to-Face Therapist Support for Depressed Primary Care Patients: Randomized Controlled Trial
Source: J Med Internet Res. 2013 Aug 5;15(8):e153. doi: 10.2196/jmir.2714 (PMC3742404; doi:10.2196/jmir.2714)
Supplement: Supplementary file 3 [file jmir_v15i8e153_app3.pdf]

Participation in a research project:

INTERNET-BASED COGNITIVE BEHAVIORAL THERAPY FOR DEPRESSIVE SYMPTOMS.

Background and purpose

This is an invitation to participate in a study as part of the BlueMood-project at the University of Tromsø. The aim of the project is to reduce depressive symptoms and to improve your mental health by using an Internet-based treatment program combined with follow-up sessions with a psychologist. We wish to convey that the problems you are encountering are common, and we would like to inform you that there are good aids and techniques you can use in order to handle them.

What does the study comprise?

If you decide to participate in this project, it will comprise the following:

1. You will meet a general practitioner or a psychologist for a preliminary consultation. This is to ensure that the program will suit your needs. During this consultation you will be asked to fill out a questionnaire concerning your thoughts and feelings.
2. You will then gain access to an Internet-based program called MoodGYM. You can log on to MoodGYM at any time. If you use the program for 45 minutes a week, it will take about six weeks to complete it.
3. During the six weeks that you are working with the MoodGYM program, you will also receive weekly follow-up consultations of 15-20 minutes with a psychologist. Questions about and experiences with the program, as well as other issues on your mind, can be discussed during these consultations.
4. At the last follow-up consultation you will again be asked to fill out a questionnaire concerning your thoughts and feelings, and your satisfaction with the treatment program.
5. Six months after the completion of the treatment, you will receive another questionnaire by e-mail.
6. The commencement of treatment will occur at somewhat differing points in time for different participants. This means that some participants may have a delay of up to 6 weeks before they start treatment. Being on a waitlist for other treatments, such as treatment from a psychologist, does not exclude you from participating in the project.

If you would like to participate, please fill in the form below.

Possible advantages and disadvantages

Previous studies of MoodGYM have not caused any discomfort or disadvantages for participants. Research has shown that Internet-based programs such as the MoodGYM, can help reduce symptoms of anxiety and depression.

What happens to your personal information?

Your personal information will only be used as described under purpose of the study. All information will be treated without names or other recognizable information. Your name and personal information is linked to a code (an ID-number). Only authorized personnel will have access to the list linking names and ID-numbers. This list is kept separate from the questionnaires. When the results of the study are published, it will be impossible to identify you.

Voluntary participation

It is voluntary to participate in this study. You are free to withdraw from participation at any time without giving an explanation. If you wish to participate please sign the declaration of consent below. If you concur now, you will still be able to withdraw without any consequences. If you would like to withdraw later on, or if you have questions concerning the study, please contact Dr Nils Kolstrup,

nils.kolstrup@uit.no

Background and purpose of the project in more detail

The BlueMood-project is carried-out in the context of the introduction of a new Internet-based program (MoodGYM), designed for improving mental health and reducing mild to moderate symptoms of depression. Research has shown that Internet-based programs such as the MoodGYM, can be of good help for those struggling with mild to moderate depressive symptoms. The program contains useful information, and teaches skills on how to cope with difficult situations and how to improve your mood. Research has shown that many people struggle with difficult thoughts and feelings, and that about 24 % of Norwegian women and 10 % of Norwegian men will experience a depressive episode during their lifetime. Most individuals with psychological problems receive treatment in general practice. The current study is part of a more comprehensive investigation where MoodGYM will be tested as a tool in the treatment of depression in general practice.

Before the Internet-based program MoodGYM can be available to more patients in general practice, we wish to find out how people experience the program and how short follow-up consultations can supplement the treatment. The results of this study will thus be of great importance as part of the preparation for a project in general practice. Your participation can contribute to a quality assurance in the development of Internet-based resources for treating depression and improving mental health. This research takes place within the municipality of Tromsø.

Personal information protection

Your name, telephone number, e-mail and ID-number constitute the information that is registered. The ID-number is the only thing that is stored with the questionnaires. Other information will be stored separately with the project's secretary, and will be shredded after the last follow-up.

Right to access and deletion of your personal information

If you concur to participate in this study, you have access to all the information registered about you. If you withdraw, you can insist on having all your personal information deleted, unless the information is already used in analyses or in scientific publications.

Information about the outcome of the study

You are entitled to receive information about the outcome of the study. You can get this from the project's secretary:

BlueMood-Prosjektet - allmennpraksis
Faculty of Health Sciences
Department of Psychology
University of Tromsø
9037 Tromsø

Consent to participation in study.

I am willing to participate in the study:

ID-number:

Name:_______________________________________

E-mail:_____________________________________

Mobile phone: ____________________________________

(Signature by participant, date)

I confirm that I have given information about this study.

(Signature by project manager, date)
